# Cation-mediated optical resolution and anticancer activity of chiral polyoxometalates built from entirely achiral building blocks†

**DOI:** 10.1039/c5sc04408a

**Published:** 2016-03-09

**Authors:** Zhi-Ming Zhang, Xiaopin Duan, Shuang Yao, Zhishu Wang, Zekai Lin, Yang-Guang Li, La-Sheng Long, En-Bo Wang, Wenbin Lin

**Affiliations:** a Key Laboratory of Polyoxometalate Science of Ministry of Education, Faculty of Chemistry, Northeast Normal University Ren Min Street No. 5268 Changchun Jilin 130024 P. R. China wangeb889@nenu.edu.cn; b Department of Chemistry, University of Chicago 929 E. 57th Street Chicago Illinois 60637 USA wenbinlin@uchicago.edu; c Collaborative Innovation Center of Chemistry for Energy Materials, Xiamen University Xiamen 361005 P. R. China

## Abstract

We report the crystallization of homochiral polyoxometalate (POM) macroanions {CoSb_6_O_4_(H_2_O)_3_[Co(hmta)SbW_8_O_31_]_3_}^15−^ (1, hmta = hexamethylenetetramine) *via* the counter cation-mediated chiral symmetry breaking and asymmetric autocatalytic processes. In the presence of low Co^2+^ concentrations both Δ- and Λ-enantiomers of 1 formed in the reaction, crystallizing into the racemic crystal *rac*-1. At a high Co^2+^ concentration, the polyoxoanion enantiomers showed a high level of chiral recognition *via* H-bonding interactions to crystallize into enantiopure crystals of Δ- or Λ-[Co(H_2_O)_6_{CoSb_6_O_4_(H_2_O)_3_[Co(hmta)SbW_8_O_31_]_3_}]^13−^. During crystallization, a microscale symmetry-breaking event and a nonlinear asymmetric autocatalysis process make the enantiomers crystallize in different batches, which provides an opportunity to isolate the homochiral bulk materials. The defined structures of the racemic and homochiral crystals thus provide a molecular-level illustration that H-bonding interactions are responsible for such high-level chiral recognition, in a process similar to the supramolecular chirality frequently observed in biology. These POM macroanions showed a high cytotoxicity against various cancer cells, particularly ovarian cancer cells. The antitumor activity of these compounds resulted at least in part from the activation of the apoptotic pathways, as shown by the flow cytometry, Annexin V staining, DNA ladder, and TUNEL assay, likely by blocking the cell cycle and complexing with proteins in cells. The POM macroanions reported herein provide promising and novel antitumor agents for the potential treatment of various cancers.

## Abstract 2

Homochiral polyoxometalates were optically resolved via the counter cation-mediated breaking of chiral symmetry, and showed potent cytotoxicity against various cancer cells by activating apoptotic pathways.

## Graphical abstract

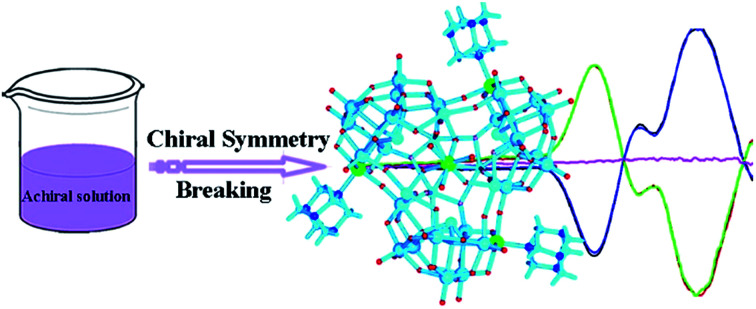

## Introduction

Chirality plays a critical role in biology and is also important for the functions of many materials.^1–3^ In biological systems, homochirality is an essential feature of basic building blocks, such as amino acids and sugars, which are distinctly left- and right-handed.^4,5^ Although the origin of homochirality is firmly established in biological systems, the spontaneous assembly of enantiopure materials from achiral precursors still represents a great challenge to chemists and material scientists.^6,7^

Polyoxometalates (POMs), a class of metal–oxo clusters with oxygen-rich surfaces, have been extensively explored for applications in biology, magnetism, catalysis, and material science.^8^ The introduction of chirality into POMs or POM–organic hybrid compounds can endow further functionality for potential applications in nonlinear optics, enantioselective catalysis, chiral medicine, and chiral magnetism.^9–14^ Chiral ligands are typically used as structure-directing agents to facilitate the synthesis of chiral POM materials.^11–13^ A more versatile approach towards such a synthesis would use completely achiral precursors to construct chiral POM skeletons *via* bond length alteration, structure distortion, formation of lacunae, replacement with other metals, or decorating with organic ligands to remove the inversion and/or mirror symmetry in POMs.^9,14^ However, bulk materials generally contain a racemic mixture of chiral POMs or POM materials. Achieving homochirality from totally achiral precursors is still a significant challenge in the laboratory.^15^

Among numerous potential applications, POMs have been reported to possess promising antibacterial, antiviral, and anticancer activities, which may open the way toward new and affordable therapeutic strategies for various human diseases.^16^ Several POMs have been recognized as potential antitumor agents;^17^ in particular, [NH_3_Pr^i^]_6_[Mo_7_O_24_]·3H_2_O (PM-8) was found to exhibit antitumor activity against multiple cancers *in vitro* and *in vivo*.^18^

We observed that chiral recognition between POM macroanions and Co(H_2_O)_6_^2+^ counter cations *via* hydrogen bonds led to chiral symmetry breaking, affording homochiral crystalline materials. These POM macroanions also exhibited potent antitumor activities against various human cancer cells, resulting from the activation of apoptotic pathways by blocking the cell cycle and complexing with proteins in cells.

## Results and discussion

### Synthesis

The chiral POM-based molecular capsule {CoSb_6_O_4_(H_2_O)_3_[Co(hmta)SbW_8_O_31_]_3_}^15−^ (hmta = hexamethylenetetramine) was assembled by treating the achiral polyoxoanion [NaSb_9_W_21_O_86_]^18−^ ({Sb_9_W_21_}) with Co^2+^ ions and hmta molecules in aqueous solution. In the synthesis, the trimeric {Sb_9_W_21_} ion with *C*_3h_ symmetry (Fig. S1a†), comprising three {SbW_7_O_24_} ({SbW_7_}) units surrounding a central {NaSb_6_O_14_} core, was used as the starting material. The resulting POM macroanion contains three [β-Co(hmta)SbW_8_O_32_] units surrounding a central {CoSb_6_O_4_(H_2_O)_3_} core, affording a trimeric capsule-like molecule with *C*_3_ symmetry. Both (NH_4_)_8_[Δ-Co(H_2_O)_6_{CoSb_6_O_4_(H_2_O)_3_[Co(hmta)SbW_8_O_31_]_3_}]·(Hhmta)_5_·29H_2_O (Δ-2) and (NH_4_)_8_[Λ-Co(H_2_O)_6_{CoSb_6_O_4_(H_2_O)_3_[Co(hmta)SbW_8_O_31_]_3_}]·(Hhmta)_5_·29H_2_O (Λ-2) enantiomers formed during the assembly process, resulting in a racemic solution that crystallized into racemic crystals of (NH_4_)_9_{CoSb_6_O_4_(H_2_O)_3_[Co(hmta)SbW_8_O_31_]_3_}·Cl·(Hhmta)_7_·33H_2_O (1) at low Co^2+^ concentrations (*ca.* 46 mM).

Crucial to the reaction was the pH value, which was in the 7.5–8.5 range at the beginning of the reaction and decreased to 7.0–7.6 after two hours. The hmta molecules not only acted as the coordinating ligands but also served as a base to control the pH value of the reaction system. If other bases such as NH_3_·H_2_O, NaOH, and KOH were used to replace hmta in the reaction, the capsule-type POMs could not be isolated. In the synthesis, by increasing the Co^2+^ concentration, the capsules underwent a completely enantioselective self-resolution; they self-assembled into individual single crystals composed of the same enantiomers but did not form racemic single crystals comprising opposite enantiomers. Strikingly, the homochiral crystallization process led to the same handedness of individual crystals in each batch, affording the homochiral bulk materials [Δ-Co(H_2_O)_6_{CoSb_6_O_4_(H_2_O)_3_[Co(hmta)SbW_8_O_31_]_3_}]^13−^ (Δ-2) and [Λ-Co(H_2_O)_6_{CoSb_6_O_4_(H_2_O)_3_[Co(hmta)SbW_8_O_31_]_3_}]^13−^ (Λ-2).

### Structure

**Fig. 1:**
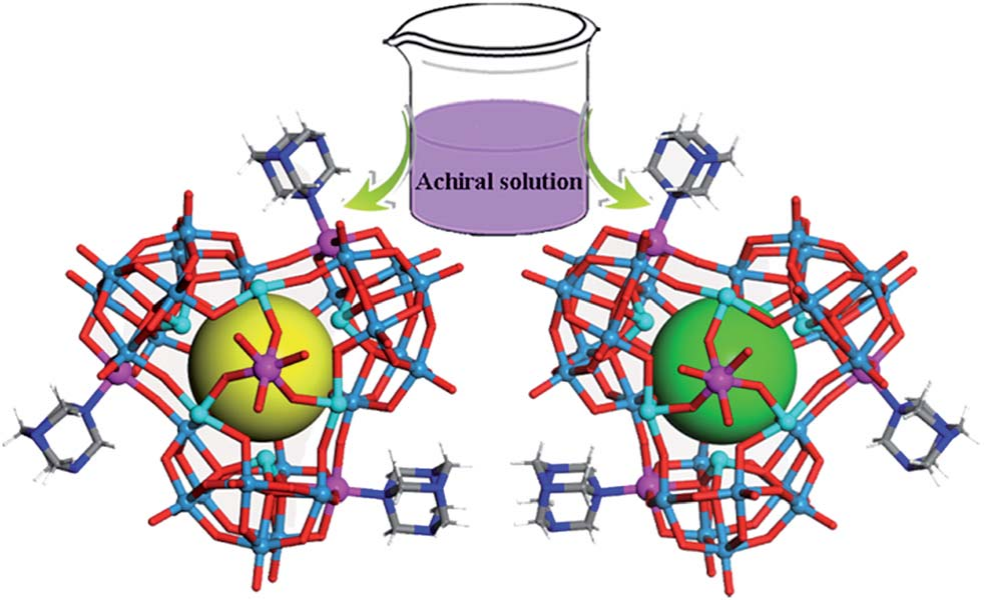
Schematic showing the spontaneous resolution of enantiopure molecular capsules from achiral building blocks. W, light blue; Sb, cyan; Co, purple; N, blue; O, red; C, light grey; H, white.

Compound 1 crystallizes in the achiral monoclinic *P*2_1_/*c* space group, whereas compound 2 crystallizes in the cubic chiral space group *P*2_1_3. Single-crystal X-ray diffraction analyses revealed that compounds 1 and 2 both contain a screw propeller-like cluster {CoSb_6_O_4_(H_2_O)_3_[β-Co(hmta)SbW_8_O_31_]_3_}^15−^, which is built from a trimeric capsule-type polyoxotungstate and three hmta ligands (Fig. 1 and S1b†). The capsule-type POM {CoSb_6_O_4_(H_2_O)_3_[β-Co(hmta)SbW_8_O_31_]_3_} consists of a cryptate polyoxoanion {Sb_6_O_4_(β-SbW_8_CoO_31_)_3_} that is composed of three tetravacant tungstoantimonates [β-SbW_8_O_31_] (Fig. S2 and S3†). In the POM family, trivacant Keggin tungstoantimonate {SbW_9_O_33_} is a common structural motif that is used to construct other POM compounds including dimeric systems and 1-D and 2-D polymeric structures.^19^ The pentavacant {SbW_7_} unit is also observed in the cryptate polyoxoanion {Sb_9_W_21_}.^20^ However, trimeric capsule-type tungstoantimonates based on tetravacant [β-SbW_8_O_31_] units in 1 and 2 have not been reported in the literature.

**Fig. 2:**
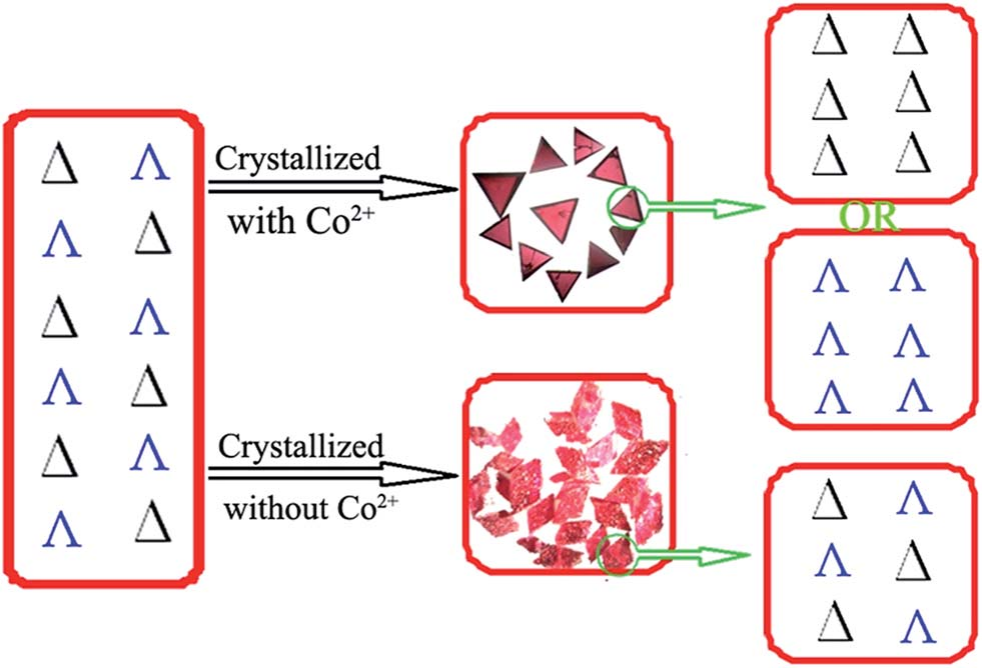
Schematic view of the chiral recognition during crystallization of the racemic solution to the homochiral crystal with Co^2+^-mediated interactions (top), or to the racemic crystal (bottom).

Each tetravacant [β-SbW_8_O_31_] unit captures one Co^2+^ ion to form a mono-cobalt-substituted trivacant Keggin-type polyoxoanion [β-SbW_8_CoO_31_], which is fused together by W–O–Co bonds to afford a triangular polyoxoanion [β-SbW_8_CoO_31_]_3_ (Fig. S4 and S5†). The triangular polyoxoanion [β-SbW_8_CoO_31_]_3_ combines with six Sb^3+^ ions to form a cryptate-type polyoxoanion {Sb_6_O_4_(β-SbW_8_CoO_31_)_3_}. In the cryptate-type polyoxoanion, there are nine Sb^3+^ centers in total, which can be divided into three groups according to their positions and functions (Fig. S2 and S6†). The first three Sb^3+^ ions reside at the central sites of the [β-SbW_8_O_31_] units to form the tetravacant β-Keggin structure [β-SbW_8_O_31_]; the second three Sb^3+^ ions locate at one side of the cryptate-type polyoxoanion [β-SbW_8_CoO_31_]_3_, and are fused together by a μ_3_-oxo atom to form the triangular bottom of the polyoxoanion cryptate (Fig. S6c†); the last three Sb^3+^ ions form a triangular {Sb_3_} group that caps on the cryptate-type polyoxoanion (Fig. S6b†). Furthermore, the cryptate-type polyoxoanion captures one Co^2+^ to afford a nanosized molecular capsule. The molecular capsule is further functionalized by three hmta ligands, resulting in the screw propeller-like cluster {CoSb_6_O_4_(H_2_O)_3_[β-Co(hmta)SbW_8_O_32_]_3_} (Fig. 1 and S1b†), which exhibits a 3-fold rotational axis corresponding to the *C*_3_ symmetry. In the crystallization process, the triangular cluster compounds form highly transparent and well-shaped single crystals, showing rhomboid and tetrahedral shapes for 1 and 2, respectively (Fig. 2). The bulk achiral and chiral crystals can be identified based on their shapes with the naked eye.

### Enantioselective crystallization

**Fig. 3:**
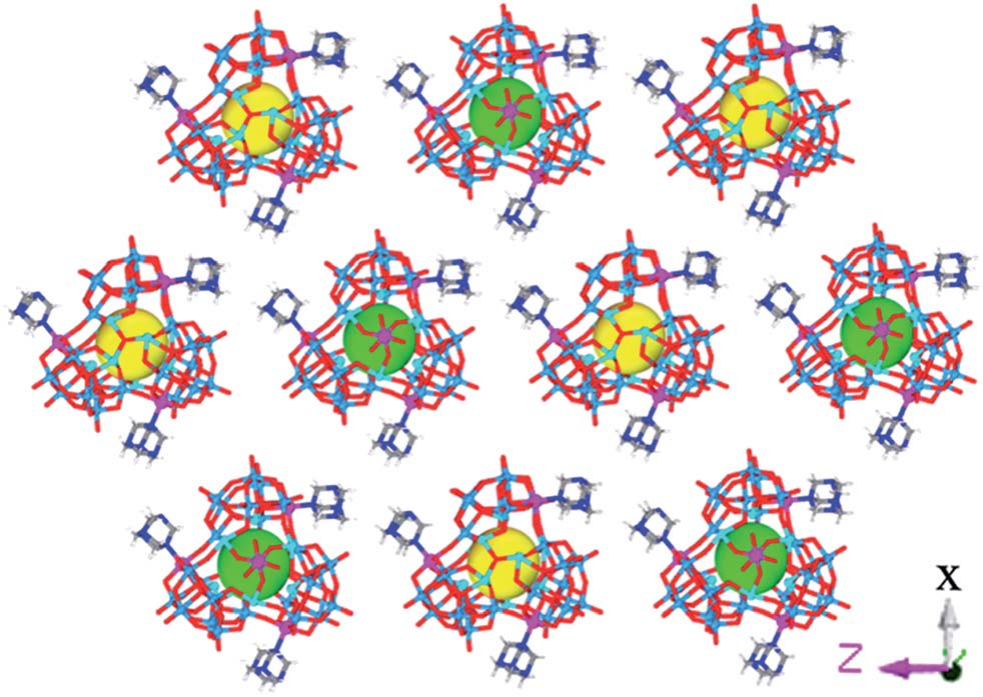
The Δ- and Λ-enantiomers arranged in the racemic crystal. Hhmta and water molecules are omitted for clarity.

**Fig. 4:**
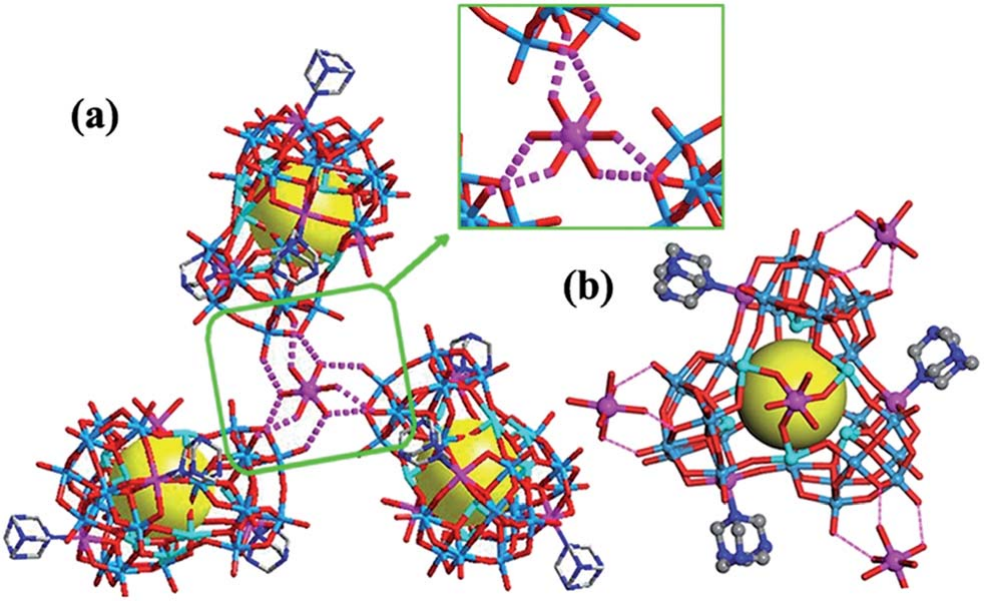
(a) The chiral Co^2+^ centre formed by coordinating three homochiral polyoxoanions *via* H-bonding interactions in Δ-2; (b) each microanion was connected to three [Co(H_2_O)_6_]^2+^ groups in this 3D structure, imparting chirality to the whole crystal. Counter Hhmta and water molecules are omitted for clarity.

**Fig. 5:**
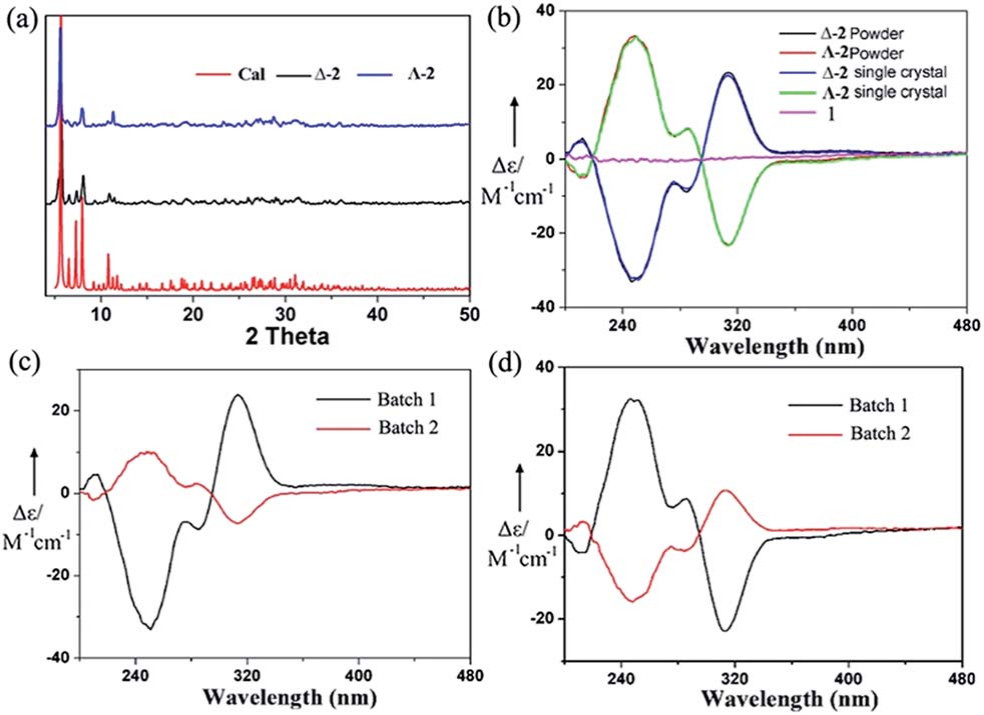
(a) Calculated and experimental PXRD patterns of Δ-2 and Λ-2; the peak positions of a simulated and experimental PXRD pattern at 20 °C are in agreement with each other; (b) CD spectra for one chiral single crystal, powder samples, and one achiral single crystal in deionized water at the same concentration (1.49 mg mL^−1^); (c) and (d) CD spectra of chiral powder samples obtained by the first crystallization and second crystallization processes; (c) shows that the Δ-type POM was isolated in the first batch and the Λ-enantiomer was isolated in the second batch, and (d) shows that the Λ-type POM was isolated in the first batch and the Δ-enantiomer was isolated in the second batch.

The chiral molecular capsule was assembled from achiral building blocks in aqueous solution. Circular dichroism (CD) studies confirmed that both enantiomers formed in the reaction. Single crystal X-ray diffraction analysis revealed that the enantiomers displayed a complete enantioselective self-resolution with the assistance of Co^2+^ ions during the crystallization process (Fig. 2). The formation of heteromeric assemblies was also observed without co-crystallization of the Co^2+^ counter cation (Fig. 3), while enantiopure crystals of Δ- or Λ-[Co(H_2_O)_6_{CoSb_6_O_4_(H_2_O)_3_[Co(hmta)SbW_8_O_31_]_3_}]^13−^ formed in the presence of high concentrations of Co^2+^ (Fig. 4). As shown in Fig. 5, the CD spectra of the chiral crystals exhibited strong Cotton effects at 314, 285, 248, and 211 nm, indicating that polyoxoanion 2 possesses optical activity in aqueous solution. This also confirmed the chiral characteristic of the anion cluster. The CD spectra of the two products were mirror images, and they possessed similar powder X-ray diffraction (PXRD) patterns (Fig. 5a), which conclusively demonstrates that they are enantiomers.

Afterward, a single crystal was selected and dissolved in deionized water with sonication. A powder sample batch randomly picked from the bulk products was also dissolved in deionized water. Held at the same concentration, the two solutions were tested by CD and UV-vis measurements, respectively (Fig. 5b and S7†). As shown in Fig. 5b, the CD spectrum of the powder sample was almost identical to that of the single crystal at the same concentration, which revealed that the bulk samples of 2 crystallized in enantiopure form. Moreover, the CD spectra of mixtures of all the starting materials in the water solution and the filtrate after the reaction did not exhibit any peaks (Fig. 5b and S8d†). After collecting the first batch of large tetrahedral crystals from the solution and allowing the water in the filtrate to slowly evaporate, another batch of small triangular crystals could be obtained. CD spectra indicated that the individual crystals obtained from the second batch exhibited the opposite chirality. However, the decrease of Cotton effects in the CD spectra of the bulk powder samples showed their reduced optical purity (Fig. 5c and d). These results indicated that the two enantiomers both formed in the reaction but crystallized out of the solution at different crystallization rates. We can conclude that chiral symmetry breaking and chiral autocatalysis occurred during the crystallization process.^21^

The nucleation of a monochiral crystal is a symmetry-breaking event on the microscale, and the nonlinear autocatalytic dynamics of secondary nucleation leads to chiral amplification and the eventual production of enantiopure crystals. In this process, the entire chirality should be influenced by the chirality of the primary nucleus. The probability of attaining a left- or right-handed chiral form should be random in a symmetry-breaking event in the absence of any chiral influence. To this end, we carried out 60 crystallization experiments and performed CD studies on each of the first batch crystals. We found that 33 of these crystallizations gave Δ-enantiomers in their first batches, while 27 of these crystallizations afforded Λ-enantiomers. Slow crystallization favored the formation of a homochiral high-nuclearity polytungstate assembly from achiral precursors. CD spectroscopy and single X-ray analysis showed that the chiral molecular capsule still possessed a high-level of self-recognition during the fast crystallization process with a high concentration of Co^2+^ ions, leading to homochiral individual crystals (Fig. S8†). However, the decrease of the CD peaks of the bulk samples compared to that of the single crystal at the same concentration indicated that some crystals with opposite handedness were formed from the solution in a fast crystallization process, resulting in a lower optical purity.

Achiral single crystal samples with a rhomboid shape (1) composed of the same polyoxoanions could be isolated by decreasing the concentration of Co^2+^ in the reaction (to ≤46 mM). No Cotton effects were observed on the CD spectra of individual single crystals (Fig. 5b). The defined structures of the racemic and homochiral crystals thus provided a chance to study delicate interactions at the molecular-level for chiral recognition and propagation. As shown in Fig. 4, the co-crystallization of anion clusters and [Co(H_2_O)_6_]^2+^ groups resulted in homochiral crystals. Each [Co(H_2_O)_6_]^2+^ group was coordinated with three homochiral microanions through H-bonding interactions, with the typical H-bond length in the range of 2.6–2.9 Å. The three homochiral microanions could be regarded as three bidentate ligands coordinated with the central [Co(H_2_O)_6_]^2+^ group to form the shape of a Δ or Λ propeller. The chiral microanion self-sorted *via* the chiral [Co(H_2_O)_6_]^2+^ groups into homochiral materials (Fig. 4). This illustrated that H-bonding interactions are responsible for such high-level chiral recognition, in a process similar to the supramolecular chirality frequently observed in biology.^22^ In comparison, without the co-crystallization of Co^2+^ ions, two enantiomers were arranged alternately, resulting in racemic crystals of compound 1 (Fig. 3).

### Stability

The stability of the POMs in aqueous solutions of different pH values was investigated by UV-vis spectroscopy and cyclic voltammetry (CV). Compound 2 was dissolved in aqueous solutions of pH 5–9 and kept at room temperature. The UV-vis spectra of these solutions were taken every hour. No changes were observed in the UV-vis spectra at pH 5–8 after a 6-hour incubation, suggesting that 2 is stable in pH 5–8 buffer solutions (Fig. S12a–d†). In the pH = 9 buffer solution, the absorbance of 2 decreased over time (Fig. S12e†), indicating that 2 is unstable at that pH. To further confirm the stability of the POMs, the CV behaviors of 2 in pH 5–8 buffer solutions were obtained. No obvious changes in the CV characteristics were observed during 6-hour treatments (Fig. S12f–i†). We further studied the solution structure by ESI-MS. After compound 1 was dissolved in a pH 7 buffer solution and aged for 24 hours, ESI-MS spectra confirmed the existence of 1 (Fig. S13 and Table S2†), which was in agreement with the solid state structures from X-ray diffraction experiments. These results confirm that the POMs are structurally stable in pH 5–8 aqueous solutions.

### Cytotoxicity

In order to determine whether the present POMs possess antitumor activities and on which cell lines they are more effective, we studied their cytotoxicity against eight cancer cell lines, including four ovarian cancer cell lines, two colon cancer cell lines, one non-small lung cancer cell line and one breast cancer cell line, by the (3-(4,5-dimethylthiazol-2-yl)-5-(3-carboxymethoxyphenyl)-2-(4-sulfophenyl)-2*H*-tetrazolium) (MTS) assay (Fig. S14, Tables 1, S3 and S4†). The cytotoxicity of *rac*-1 was similar to both Δ-2 and Λ-2, and significantly higher than that of {Sb_9_W_21_} on all tested cell lines. Specifically, 1 and 2 showed a higher cytotoxicity on A2780, A2780cisR, and OVCAR-3 cells than on other cells, which meant that they are very effective on ovarian cancer cell lines. The IC_50_ values (the concentration for a 50% growth inhibition) of 1 on these three cell lines were 0.77 ± 0.01, 4.35 ± 0.20, and 1.78 ± 0.07 μM, respectively. In addition, hmta and NH_4_Cl showed no cytotoxicity on all tested cell lines, even at very high concentrations (>500 and 900 μM, respectively). Although CoCl_2_ exhibited some cytotoxicity on A2780 and A2780cisR cells, its IC_50_ values (122.7 ± 3.21 and 114.6 ± 9.75 μM, respectively) were significantly higher than those of 1 and {Sb_9_W_21_}. These results indicated that the higher cytotoxicity of 1 and 2 over {Sb_9_W_21_} was derived from POM assemblies, not the addition of other components. We also determined the cytotoxicity of POMs against normal cell line HEK-293 cells. The IC_50_ value of POMs on HEK-293 cells is around 16 μM, which is ∼20-, 8.9- and 3.6-fold higher than that on A2780, OVCAR-3 and A2780cisR cells, respectively (Fig. S16 and Table S6†). These results demonstrated the potential use of POMs for the treatment of ovarian cancer.

**Table 1:** IC_50_ values (μM) of POMs against A2780, A2780cisR, and OVCAR-3 cells after a 72-hour incubation, as determined by the MTS assay. Data are expressed as means ± S.D. (*n* = 3)

	A2780 cells	A2780cisR cells	OVCAR-3 cells
1	0.77 ± 0.01	4.35 ± 0.20	1.78 ± 0.07
Δ-2	0.78 ± 0.01	4.51 ± 0.25	1.81 ± 0.01
Λ-2	0.80 ± 0.03	4.42 ± 0.11	1.80 ± 0.08
{Sb_9_W_21_}	4.44 ± 0.13	29.02 ± 1.31	8.80 ± 0.18

### Cellular uptake

**Fig. 6:**
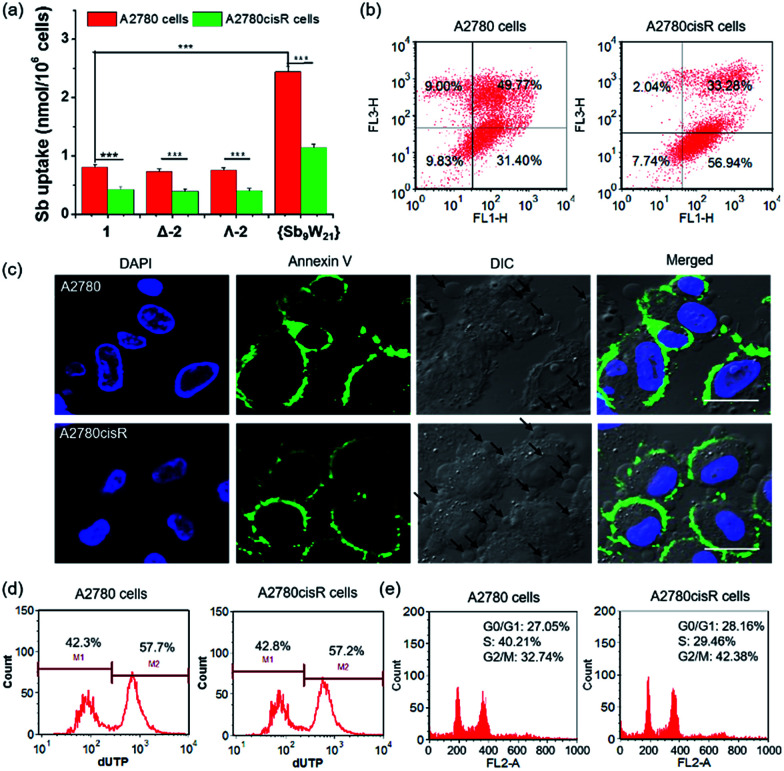
(a) Uptake of Sb by A2780 and A2780cisR cells incubated with POMs for 4 hours (***P < 0.001); (b) quantitative analysis of apoptosis induced by 1 in A2780 and A2780cisR cells; (c) CLSM images showing cell apoptosis induced by 1 in A2780 and A2780cisR cells. The apoptotic bodies are labeled with black arrows. Scale bars: 20 μm; (d) flow cytometric analysis of the apoptotic and non-apoptotic populations of A2780 and A2780cisR cells treated with 1 by a TUNEL assay; (e) cell cycle analysis of A2780 and A2780cisR cells incubated with 1 for 24 hours.

Encouraged by the high cytotoxicity of POMs against A2780 and A2780cisR cells, the cellular uptake of POMs on both cell lines was investigated by directly measuring the metal content in the cells using inductively coupled plasma-mass spectrometry (ICP-MS, Agilent technologies, Santa Clara, CA) (Fig. 6a and S17†). After incubation for 4 hours at an equivalent concentration of 20 μM, the Sb content in A2780 cells was 0.78 ± 0.04, 0.74 ± 0.04, 0.75 ± 0.04, and 2.44 ± 0.08 nmol per 10^6^ cells, and the W content was 2.02 ± 0.10, 2.08 ± 0.11, 2.03 ± 0.10, and 5.74 ± 0.29 nmol per 10^6^ cells for 1, Δ-2, Λ-2, and {Sb_9_W_21_}, respectively. In addition, the Co content in A2780 cells was 0.36 ± 0.02, 0.40 ± 0.03, and 0.41 ± 0.03 nmol per 10^6^ cells for 1, Δ-2, and Λ-2, respectively. These results indicated that the uptake of 1 is similar to 2, and three times less than that of {Sb_9_W_21_}. The molar ratio of internalized Sb to W was 0.38 ± 0.01, 0.36 ± 0.02, and 0.37 ± 0.01 (around 9 : 24) for 1, Δ-2, and Λ-2, and 0.43 ± 0.01 (approximately 9 : 21) for {Sb_9_W_21_}, and the molar ratio of internalized Co to Sb was 0.46 ± 0.03 (around 4 : 9) for 1 and 0.54 ± 0.01 and 0.55 ± 0.02 (roughly 5 : 9) for Δ-2 and Λ-2, respectively. These ratios did not significantly deviate from those expected for the POMs, suggesting that the POMs and {Sb_9_W_21_} remain intact upon cellular uptake. The same molar ratio on A2780cisR cells was also observed, except that the uptake of the title compounds and {Sb_9_W_21_} on A2780cisR cells was about two times less than on A2780 cells, which may explain the lower cytotoxicity of POMs on A2780cisR cells than on A2780 cells. The cellular uptake of POMs on A2780 cells in the presence or absence of 10% FBS was also determined (Fig. S18†). No difference in the cellular uptake was observed for the POMs incubated with or without FBS, indicating that FBS in the cell medium did not block the uptake of POMs.

### Apoptosis analysis

It has been reported that POMs can exert cytotoxicity by inducing apoptosis of cancer cells.^17*b*,18*a*^ Thus, a flow cytometry assay was performed to see whether the present POMs can induce apoptosis on A2780 and A2780cisR cells. As shown in Fig. 6b and S19,†1, Δ-2, and Λ-2 induced very high levels of cell apoptosis, resulting in 81.17%, 80.96%, and 80.36% apoptotic cells in A2780 cells, and 90.22%, 88.68%, and 85.16% apoptotic cells in A2780cisR cells, respectively. However, {Sb_9_W_21_} at the same concentration barely induced cell apoptosis on either cell line. These results suggest that the antitumor activity of POMs is attributable at least in part to the activation of the apoptotic pathway in cancer cells.

The cell apoptosis induced by POMs was also confirmed by confocal laser scanning microscopy (CLSM) (Fig. 6c and S20†). Most of the cells treated with POMs appeared to have apoptotic morphologies, as shown by membrane blebbing and the appearance of the membrane-associated apoptotic bodies. In addition, the presence of bright green fluorescence from Annexin V in the cells treated with POMs confirmed that POMs could successfully induce cancer cell apoptosis. On the contrary, an apoptotic morphology and green fluorescence were not observed in cells treated with {Sb_9_W_21_}, indicating that {Sb_9_W_21_} could not induce cell apoptosis at the same concentration. These results were consistent with the quantitative results of flow cytometry.

DNA fragmentation provided additional support for apoptosis. During apoptosis, activated nucleases degrade the higher order chromatin structure of DNA into fragments, which can be extracted from cells and visualized by gel electrophoresis followed by ethidium bromide staining. As shown in Fig. S21,† the presence of the characteristic DNA ladder in lanes 3–5 suggested that POMs can induce significant apoptosis in both cell lines. In contrast, the absence of the DNA ladder in lane 6 indicated that {Sb_9_W_21_} could not induce apoptosis in these cell lines. The DNA fragments were further quantified by a terminal deoxynucleotidyl transferase mediated dUTP nick end labeling (TUNEL) assay (Fig. 6d and S22†). POMs resulted in ∼55% of apoptotic cells in both A2780 and A2780cisR cells. In contrast, cells treated with {Sb_9_W_21_} at the same concentration did not show any apoptotic characteristics.

### Cell cycle analysis

The ability to proliferate indefinitely is one of the identifying characteristics of cancer cells, and is key to tumor growth and development. The alteration of the cell cycle induced by POMs was analyzed by determining the amount of DNA in the cells. As shown in Fig. 6e and S23,† most of the blank cells are in the G_0_/G_1_ phase, suggesting that the cells were preparing for DNA synthesis. The cell cycle significantly changed after treatment with 1, Δ-2, and Λ-2, with the ratio of the G_0_/G_1_ phase decreasing by 52.2%, 46.2%, and 45.3% in A2780 cells, and 33.9%, 37.1%, and 39.3% in A2780cisR cells, respectively. Likewise, the percentage of the G_2_/M phase increased 2.57-, 2.01-, and 2.26-fold in A2780 cells, and 1.85-, 1.27-, and 1.96-fold in A2780cisR cell, respectively, after treatment with 1, Δ-2, and Λ-2. These results indicated that POMs can inhibit cancer cell proliferation by blocking the cell cycle. However, cells treated with {Sb_9_W_21_} exhibited a cell cycle similar to the control cells, suggesting that {Sb_9_W_21_} could not change the cell cycle at a comparable concentration.

### Protein binding studies

When drugs are bound to proteins, the resulting protection from metabolic degradation can prolong their activity. The proportion of drug molecules bound to the protein depends on the total drug concentration and the drugs' affinity to the protein. The distribution and metabolism of many biologically active compounds are correlated with their affinities to serum albumin, the most abundant protein in blood plasma, accounting for about 60% of its total protein. Thus, an investigation of the interaction between these compounds and serum albumin can provide important information on their potential as therapeutic drugs.^23^

The interaction of POMs with bovine serum albumin (BSA) was investigated using multi-spectroscopic methods. UV-vis absorption measurements indicate the structure and the conformation of the complex. As shown in Fig. S24 and Table S7,† BSA displayed a strong absorption peak at 204 nm, which represents the content of an α-helix structure. After the addition of POMs, the peak intensity decreased, which was accompanied by a bathochromic shift from 204 nm to 210 nm. The absorbance at 280 nm, mainly coming from tryptophan, tyrosine, and phenylalanine, increased in the presence of increasing amounts of POMs. The changes in the characteristic absorption indicated that POMs can interact with BSA, and this interaction may change the structure of BSA by decreasing the number of α-helix structures. Interestingly, the increase in the absorbance at 280 nm of BSA titrated with {Sb_9_W_21_} (about 18%) was much lower than when it was titrated with POMs (approximately 35%), which indicated that {Sb_9_W_21_} may form fewer complexes with BSA than POMs do.

**Fig. 7:**
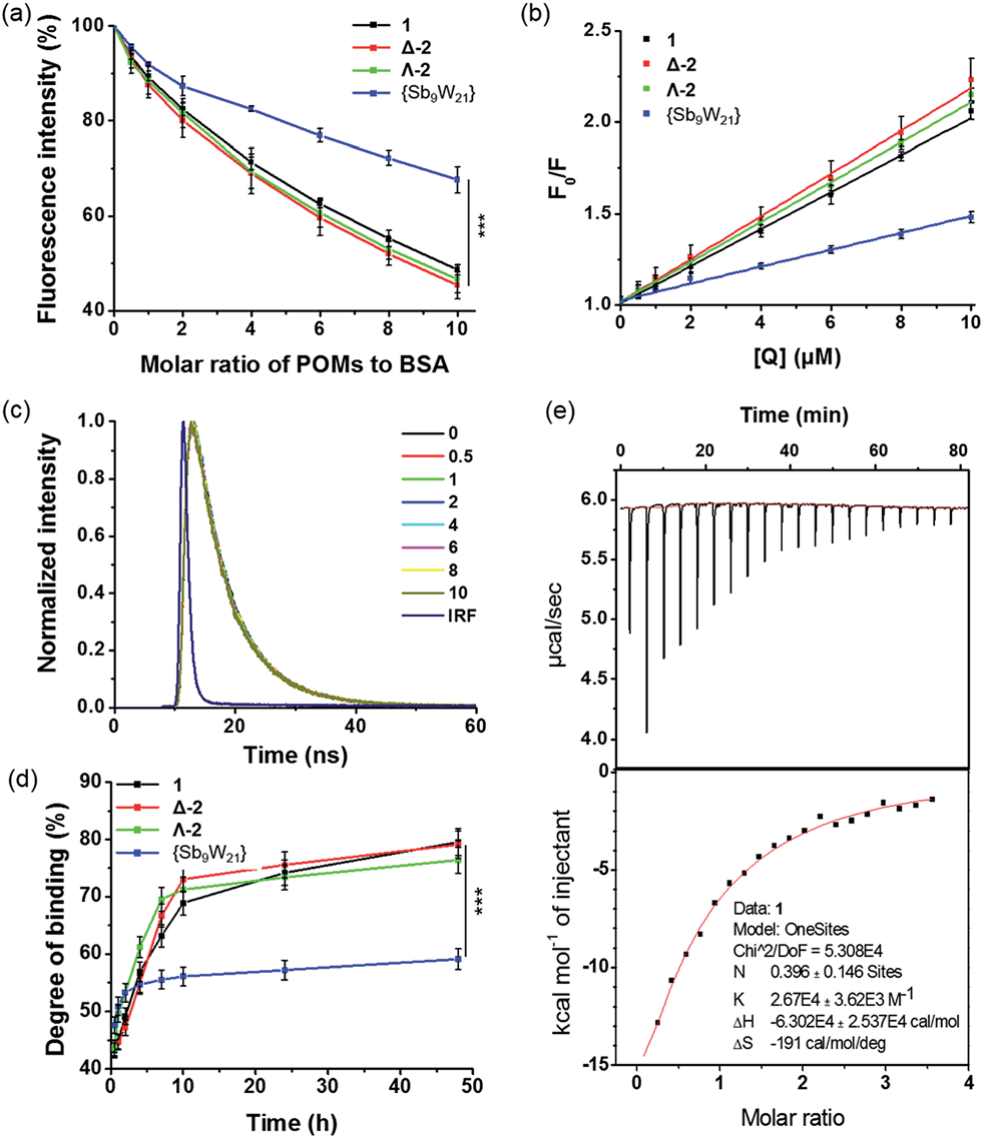
(a) Decrease in the fluorescence of BSA in the presence of increasing amounts of POMs (***P < 0.001); (b) Stern–Völmer curves of the POMs–BSA systems; (c) time-resolved fluorescence decay traces of BSA titrated with 1, with a molar ratio of 1 to BSA of 0–10. “IRF” in the figure stands for Instrument Response Function; (d) binding of POMs to BSA at variable reaction times (***P < 0.001); (e) calorimetric data for the titration of BSA with 1. The binding isotherm (heat change *vs.* POM/BSA molar ratio) was obtained from the integration of raw data and was fitted to a “one-site” model.

An intrinsic fluorescence study was performed to evaluate the changes in structure that were caused by the reaction of BSA with POMs. Fig. S25† shows a conspicuous change in the fluorescence emission spectra of BSA with the addition of various amounts of POMs. In the absence of POMs, the relative fluorescence intensity of BSA was about 70; in the presence of POMs, the relative fluorescence intensities obviously decreased to 35, 31, 34, and 48 for 1, Δ-2, Λ-2, and {Sb_9_W_21_}, respectively, which were equivalent to reductions of 51.4%, 54.5%, 53.4%, and 32.4%, respectively (Fig. 7a). These phenomena and analyses suggested that POMs could bind to BSA, and that the binding induces some changes in the microenvironment of the tryptophan residues.^24^ The lower quenching effect of {Sb_9_W_21_} was consistent with the UV results, and suggested that {Sb_9_W_21_} forms fewer complexes with BSA than POMs do.

The fluorescence quenching data were further analyzed by the Stern–Völmer equation:^25^*F*_0_/*F* = 1 + *K*_sv_[Q] = 1 + *K*_q_*τ*_0_[Q]where *F*_0_ and *F* are the fluorescence intensities without and with quenchers. *K*_q_ is the quenching rate constant of the biomolecule, *K*_sv_ is the Stern–Volmer dynamic quenching constant, *τ*_0_ is the average lifetime of the molecule without the quencher (10^−8^ s) and [Q] is the concentration of the quencher, respectively.

As shown in Fig. 7b and Table S8,† the values of *K*_q_ for 1, Δ-2, Λ-2, and {Sb_9_W_21_} were (1.02 ± 0.03) × 10^13^, (1.18 ± 0.21) × 10^13^, (1.11 ± 0.89) × 10^13^, and (0.45 ± 0.06) × 10^13^ L (mol s)^−1^, respectively, which were greater than the value of the maximum scatter collision quenching constant (2.0 × 10^10^ L (mol s)^−1^). Therefore, the quenching was more of a static quenching that was initiated by complex formation, rather than by dynamic collision.

Dynamic and static quenching mechanisms can also be distinguished by changes in the fluorescence lifetime. The lifetime decreases with the addition of quenchers for dynamic quenching, while no change is observed for static quenching. The static quenching mechanism was further confirmed by the lifetime determination, with no decrease observed for the lifetime of BSA after the addition of POMs and {Sb_9_W_21_} (Fig. 7c and S26†).

The *in vitro* binding of POMs to serum albumin was quantified by an ICP-MS assay (Fig. 7d). The binding of POMs to albumin occurred practically immediately, with about 45% of POMs bound to BSA after a 30-minute incubation. The binding degree then increased to about 70% after a 10-hour incubation and reached about 80% after 48 hours of incubation. {Sb_9_W_21_} also showed fast binding to BSA, but only 60% of {Sb_9_W_21_} was bound to BSA after 48 hours of incubation, confirming the higher affinity of POMs to BSA than {Sb_9_W_21_}.

The binding constants of POMs to BSA were determined by isothermal titration calorimetry (ITC). The binding constants for 1, Δ-2, Λ-2, and {Sb_9_W_21_} were determined to be (2.67 ± 0.36) × 10^4^, (2.31 ± 0.22) × 10^4^, (2.36 ± 0.30) × 10^4^, and (1.68 ± 0.44) × 10^4^ M^−1^, respectively. In addition, about 40% of POMs were bound to BSA, while only 13% of {Sb_9_W_21_} interacted with BSA (Fig. 7e and S27†). The protein binding studies demonstrated that POMs can bind to proteins, likely through electrostatic interactions, with a higher affinity than {Sb_9_W_21_}, which may explain their greater cytotoxicity. We believe that, after internalization, POMs affect the cellular function by binding to the proteins in the cells, thereby inducing cell apoptosis and resulting in cell death. {Sb_9_W_21_} had a lower affinity to proteins than POMs, which resulted in less cell apoptosis and a lower cytotoxicity, although it showed a higher cellular uptake than POMs.

## Conclusions

In summary, homochiral crystalline materials were synthesized from achiral precursors *via* chiral symmetry breaking and asymmetric autocatalysis. During crystallization, the enantiomers showed a high level of chiral recognition, causing them to crystallize into individual enantiopure crystals through a counterion-mediated interaction. Detailed structural analysis revealed that H-bonding interactions are responsible for such high-level chiral recognition, in a process similar to the supramolecular chirality frequently observed in biology. The successful synthesis of the POM-based materials in their homochiral forms not only provided rare examples of homochiral materials, but also suggested a new structural model for studying the chemical origins of life. In addition, the POMs synthesized here were effective against various cancer cells, especially resistant ovarian cancer cells. The POMs could block the cell cycle and complex with proteins with high affinities, thereby efficiently inducing cell apoptosis and inhibiting cell proliferation. These results suggest that they are promising antitumor agents, especially for the treatment of resistant cancers and other hard-to-treat solid tumours.

## Experimental

### Materials and instruments

All the reagents were commercially purchased and used without further purification. The (NH_4_)_18_[NaSb_9_W_21_O_86_]·24H_2_O precursor was synthesized according to the literature^20^ and characterized by IR spectroscopy. Elemental analyses of Sb, W, and Co were performed on a PLASMASPEC (I) ICP atomic emission spectrometer and Agilent 7700x ICP-MS and analyzed using an ICP-MS Mass Hunter version B01.03. IR spectra were recorded in the range 400–4000 cm^−1^ on an Alpha Centauri FTIR spectrophotometer using KBr pellets. UV-vis spectroscopy was performed with a Varian Cary 50 spectrophotometer in the range of 200–800 nm. The PXRD data were recorded on a Bruker D8 Advance diffractometer. CD spectra were measured with a Jasco model J-810 spectropolarimeter. Time-domain lifetimes were measured on a ChronosBH lifetime fluorimeter (ISS, Inc.) using Time-Correlated Single Photon Counting (TCSPC) methods. The fluorimeter contained Becker–Hickl SPC-130 detection electronics and a HPM-100-40 Hybrid PMT detector. Excitation was provided by a 280 nm nanosecond pulsed LED. Emission wavelengths were selected with interference filters (Semrock BrightlineFF01-341/LP). The Instrument Response Function (IRF) was measured to be approximately 120 ps FWHM in a 1% scattering solution of Ludox LS colloidal silica. Lifetimes were fitted using a forward convolution method in the Vinci control and analysis software.

### Synthesis

#### Synthesis of 1

(NH_4_)_18_[NaSb_9_W_21_O_86_]·24H_2_O (0.60 g, 0.085 mmol) was dissolved in distilled water (10 mL), to which solid CoCl_2_·6H_2_O (0.10 g, 0.42 mmol) was added with strong stirring. The pH value of the mixture was adjusted to 7.40 with a 1.2 M hmta solution, and then solid NaOH was added to adjust the pH value to 8.50. The resulting mixture was heated at 100 °C for 2 hours. After the reaction, the pH value was 7.50. The filtrate was kept at room temperature with slow evaporation for 6 days, resulting in big purple rhomboid shaped crystals of 1 (yield *ca.* 110 mg). Anal. found (%): Co, 2.61; Sb, 12.01; W, 47.82; calcd: Co, 2.47; Sb, 11.47; W, 46.20.

#### Synthesis of 2

(NH_4_)_18_[NaSb_9_W_21_O_86_]·24H_2_O (0.6 g, 0.085 mmol) was dissolved in distilled water (10 mL), to which solid CoCl_2_·6H_2_O (0.15 g, 0.63 mmol) was added with strong stirring. The pH value of the mixture was carefully adjusted to 7.20 with a 1.2 M hmta solution, and then solid NaOH was added in this system to adjust the pH value to 8.0. The resulting mixture was heated at 100 °C for 2 hours. After the reaction, the pH value was 7.30. The filtrate was kept at room temperature with slow evaporation for 28 days, resulting in big purple tetrahedral crystals of 2 (yield *ca.* 20 mg). After collecting these crystals, the filtrate was kept at room temperature for 3 days, resulting in another batch of small purple triangular shaped crystals (yield *ca.* 88 mg). Anal. found (%): Co, 3.07; Sb, 11.89; W, 48.08; calcd: Co, 3.17; Sb, 11.77; W, 47.39. IR (KBr pellet): *ν*_max_/cm^−1^ 3403 (m), 1634 (m), 1462 (s), 1434 (w), 1382 (w), 1312 (w), 1261 (m), 1246 (s), 1231 (m), 1149 (w), 1060 (w), 1024 (s), 1002 (m), 940 (s), 804 (s), 705 (s), 526 (w) and 460 (w).

### X-ray crystallography

The single crystal X-ray diffraction of 1 was collected with a Bruker APEX II CCD based detector at ChemMatCARS (Sector 15), Advanced Photon Source (APS), Argonne National Laboratory. The frames were integrated with the Bruker SAINT build in the APEX II software package using a narrow-frame integration algorithm, which also corrects for the Lorentz and polarization effects. The crystallographic data was performed on an Oxford Diffraction Gemini R CCD for 2. The data were collected at 293 K, and with graphite-monochromated Mo-Kα radiation (*λ* = 0.71073 Å). These structures were solved by the direct method and refined by full-matrix least squares on *F*^2^ using the SHELXL-97 software.^26^ During the refinement of compounds 1 and 2, the command ‘isor’ was used to restrain the non-H atoms with ADP and NPD problems, which led to restraint values of 322 and 143 for 1 and 2, respectively. The command ‘omit-3 50’ was used to omit the weak reflection above 50 degrees for 2. There were a number of short connections between OW(water)⋯O(POM) in the range of 2.50–2.90 Å, suggesting extensive H-bonding interactions between the lattice water molecules and POMs. All hydrogen atoms on water molecules, protonation, and the counter NH_4_^+^ were directly included in the molecular formula. Carbon and the nitrogen-bound hydrogen atoms of the hmta molecules were placed in geometrically calculated positions. In the refinement, 14 and 18 lattice water molecules were found from the Fourier maps for compounds 1 and 2, respectively. However, there were still accessible solvent voids in the crystal structure, indicating that more water molecules should exist in the structure. On the basis of TG results, there should be another 15 water molecules in the formula unit, which were directly included in the molecular formula. The crystal data and structural refinements of compounds 1 and 2 are summarized in Table S1.† CCDC reference number 1431324 for 1 and 827684 for Δ-2 contains the supplementary crystallographic data for this paper.

### Cell culture

Cisplatin-sensitive human ovarian cancer cells A2780 and cisplatin resistant human ovarian cancer cells A2780cisR were obtained from Developmental Therapeutics Core, Northwestern University. Other cancer cells, including human ovarian cancer cells OVCAR-3 and SKOV-3, human colon cancer cells HT29 and CT26, human non-small lung cancer cells A549, human breast cancer cells MCF-7, and human embryonic kidney 293 (HEK-293) cells were all obtained from the American Type Culture Collection (ATCC, Rockville, MD). A2780, A2780cisR, OVCAR-3, CT26, and A549 cells were cultured in RPMI 1640 containing 10% fetal bovine serum (FBS, Gibco, Grand Island, NY). SKOV-3 and HT29 cells were grown in McCoy's 5A containing 10% FBS. MCF-7 and HEK-293 cells were cultured in Dulbecco's Modified Eagle's Medium (DMEM) supplemented with 10% FBS. All cells were cultured in a humidified atmosphere containing 5% CO_2_ at 37 °C.

### Cytotoxicity assay

Cells seeded in 96-well plates (2 × 10^3^ cells per well) were treated with different concentrations of POMs for 72 hours, and the cell viability was then measured by MTS (Promega, Madison, WI) according to the manufacturer's instructions. At the same time, the cytotoxicity of other components was also determined and they were used as controls. IC_50_ values were calculated from curves constructed by plotting cell survival (%) *versus* drug concentration (μM).

### Cellular uptake

A2780 and A2780cisR cells seeded in 6-well plates (5 × 10^4^ cells per well) were incubated with POMs (20 μM) for 4 hours. Cells were then collected, washed with PBS, dried, and digested for metal analysis by ICP-MS. The uptake level was expressed as the amount of metal uptake associated per million cells.

### Apoptosis analysis

For the flow cytometry assay, A2780 and A2780cisR cells were treated with POMs (0.8 μM and 4.5 μM, respectively) for 24 hours. Cells were then harvested, washed with PBS, stained with Alexa Fluor 488 conjugated Annexin V and PI for 15 minutes at room temperature in the dark, and analyzed by flow cytometry.

For Annexin V staining, A2780 and A2780cisR were seeded on 10 mm^2^ glass coverslips placed in 6-well plates. After treatment with POMs for 24 hours, cells were washed with PBS, fixed with 4% paraformaldehyde, and stained with DAPI and Alexa Fluor 488 conjugated Annexin V. The cells were then observed using CLSM.

For the DNA ladder, the total DNA was extracted from A2780 and A2780cisR cells incubated with POMs using a DNA ladder isolation kit (life technologies, Grand Island, NY) and examined for DNA fragmentation with 2% (w/v) agarose gel electrophoresis at 35 V for 3 hours.

For the TUNEL assay, treated A2780 and A2780cisR cells were collected, washed with PBS, fixed with 70% ethanol at 4 °C overnight. Cells were then stained with an APO-BrdU™ TUNEL Assay Kit (Molecular Probes) according to the manufacturer's instructions, and analysed by flow cytometry.

### Cell cycle analysis

A2780 and A2780cisR cells treated with POMs for 24 hours were collected, washed with PBS, fixed with 70% ethanol at 4 °C overnight and treated with RNase A for 45 min, followed by PI staining for 30 minutes. The alteration of the cell cycle was analysed by flow cytometry.

### Protein binding studies

BSA solution (1 μM) in PBS (pH = 7.2) was titrated with POMs from 0–10 μM. After equilibration, absorption spectra measurements were carried out on a Shimadzu UV-2401 spectrophotometer at 200–350 nm. Fluorescence spectra were recorded using a Shimadzu RF-5301 spectrofluorophotometer from 300 to 400 nm at an excitation wavelength of 280 nm. Time-domain lifetimes were measured on a ChronosBH lifetime fluorimeter (ISS, Inc.).

A mixture of POMs (7.5 μM) and BSA solutions (5 mg mL^−1^) was incubated at 37 °C. Aliquots were continuously taken and ultrafiltrated through a 30 kDa cut-off filter (Millipore, Bedford, Ohio) for 15 min at 10 000 rpm. The concentration of the free POMs in the ultrafiltrate was measured by ICP-MS. The degree of binding was calculated as:(*C*_0_ − *C*_free_)/*C*_0_ × 100%where *C*_0_ and *C*_free_ are the total concentration and the concentration of the free POMs, respectively.

Calorimetric titrations were carried out on a MicroCal iTC200 (MicroCal Inc., Northampton, MA), and the data were analyzed using the Origin software. Typically, 39 μL of a POM solution (500 μM) was injected into the BSA solution (30 μM in 200 μL) over 20–24 s at 240 s intervals using a 40 μL syringe rotating at 1000 rpm. The initial delay (the hold period before injections) was set at 240 s. Before use, samples were degassed at 25 °C using the ThermoVac accessory (provided by MicroCal Inc.).

## Supplementary Material

SC-007-C5SC04408A-s001

SC-007-C5SC04408A-s002
