# Supplementary material for: Cation-mediated optical resolution and anticancer activity of chiral polyoxometalates built from entirely achiral building blocks
Source: Chem Sci. 2016 Mar 9;7(7):4220–9. doi: 10.1039/c5sc04408a (PMC6013930; doi:10.1039/c5sc04408a)
Supplement: SC-007-C5SC04408A-s001 [file SC-007-C5SC04408A-s001.pdf]

Electronic Supplementary Material (ESI) for Chemical Science.
This journal is © The Royal Society of Chemistry 2016

Supporting Information

Cation-mediated optical resolution and anticancer activity of chiral polyoxometalates built from entirely achiral building blocks

Zhi-Ming Zhang,[a,b,c,†] Xiaopin Duan,[b,†] Shuang Yao,[b] Zhishu Wang,[a] Zekai Lin,[b] Yang-Guang Li,[a] La-Sheng Long,[c] En-Bo Wang[a,]*, and Wenbin Lin[b,c,]*

[a]*Key Laboratory of Polyoxometalate Science of Ministry of Education, Faculty of Chemistry, Northeast Normal University, Ren Min Street No.5268, Changchun, Jilin 130024, (P. R. China); E-mail: wangeb889@nenu.edu.cn*

[b]*Department of Chemistry, University of Chicago, 929 E. 57th Street, Chicago, Illinois 60637, United States; E-mail: wenbinlin@uchicago.edu*

[c]*Collaborative Innovation Center of Chemistry for Energy Materials, Xiamen University, Xiamen 361005, P.R. China*

CONTENTS

Section 1 Crystal Data **S2**

Section 2 Supplementary Structural Figures **S3-S5**

Section 3 Supplementary Physical Characterizations **S6-S7**

Section 4 Stability Study **S8-S9**

Section 4 Anticancer Activity Study **S10-S17**

Section 1 Crystal Data

Table S1 Crystal Data and Structure Refinement for **1** and **2**

	**1**	**2**
Empirical formula	$C_{60}H_{235}ClCo_4N_{49}O_{133}Sb_9W_{24}$	$C_{48}H_{209}Co_5N_{40}O_{135}Sb_9W_{24}$
M	9281.05	9310.35
λ/Å	0.41328	0.71073
T/K	100(2)	293(2)
Crystal dimensions/mm	0.22 × 0.21 × 0.06	0.31 × 0.31 × 0.31
Crystal system	Monoclinic	Cubic
Space group	P2(1)/c	P2(1)3
a/Å	21.9241(13)	27.14990(10)
b/Å	26.1040(15)	27.14990(10)
c/Å	42.1665(19)	27.14990(10)
β/°	119.618(2)	90
V/Å^3	20979(2)	20012.66(13)
Z	4	4
D_c/Mg m^{-3}	3.024	3.090
μ/mm^{-1}	3.274	15.428
F(000)	17448	16908
θ Range/°	0.64–15.81	3.00–24.99
Data/restraints/parameters	50185 / 322 / 2371	11180/143/737
$R_1(I > 2\sigma(I))$ [a]	0.0454	0.0615
wR_2 (all data)[a]	0.1562	0.1240
Goodness-of-fit on F^2	0.970	1.053

[a] $R_1 = \sum||F_0|-|F_C||/\sum|F_0|$; $wR_2 = \sum[w(F_0^2-F_C^2)^2]/\sum[w(F_0^2)^2]^{1/2}$

Section 2 Supplementary Structural Figures

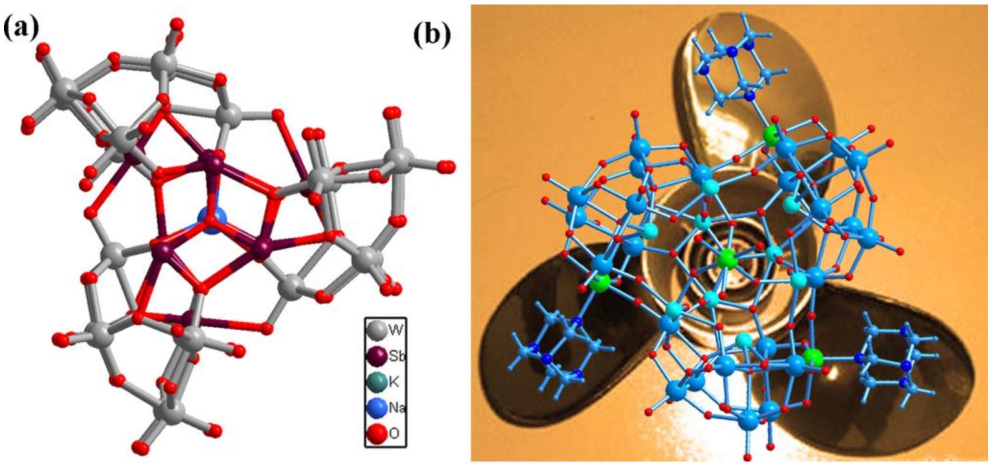

Fig. S1 (a) Ball-and-stick representation of $[NaSb_9W_{21}O_{86}]^{18-}$; (b) ball-and-stick representation of the screw propeller-like molecular capsule in **1** and **2**.

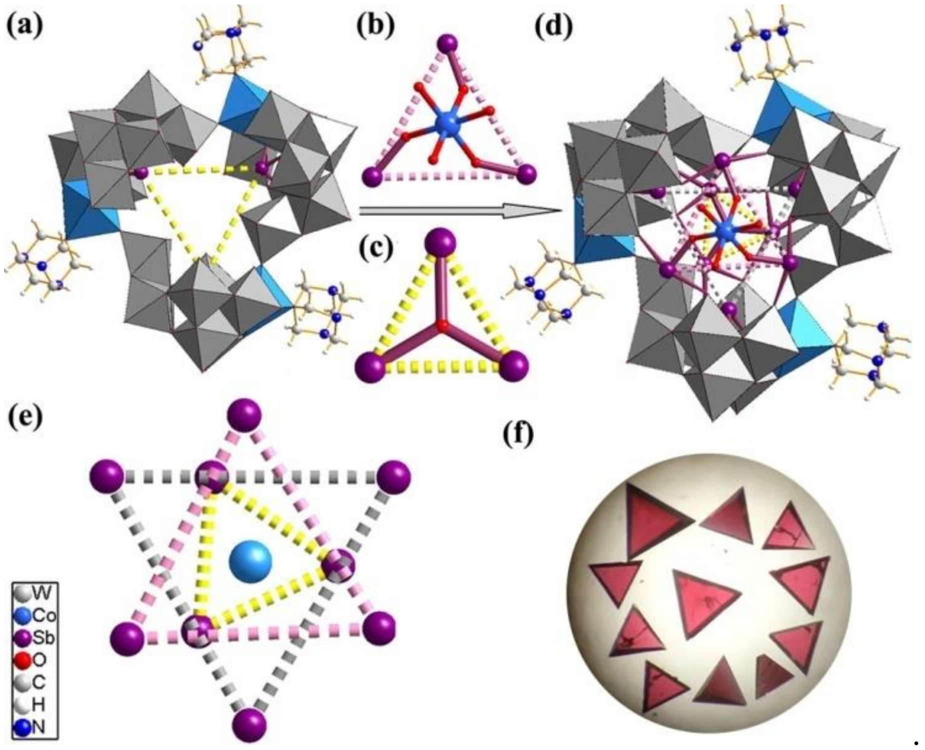

Fig. S2 (a) Triangular structure composed of three [β-Co(hmta)SbW_8O_{32}] units; (b) {Sb_3Co} group capping at the top of the capsule; (c) {Sb_3} group located at the bottom of the capsule; (d) view of the chiral polyoxotungstate cluster; (e) schematic view of the three triangular units in a chiral microanion; (f) optical micrograph of a single crystal of Λ-**2**.

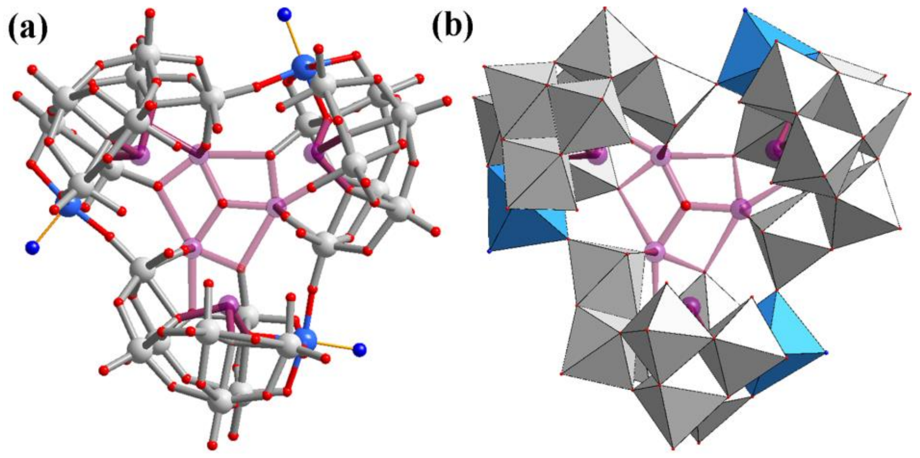

Fig. S3 The cryptate shell chiral polyoxoanion.

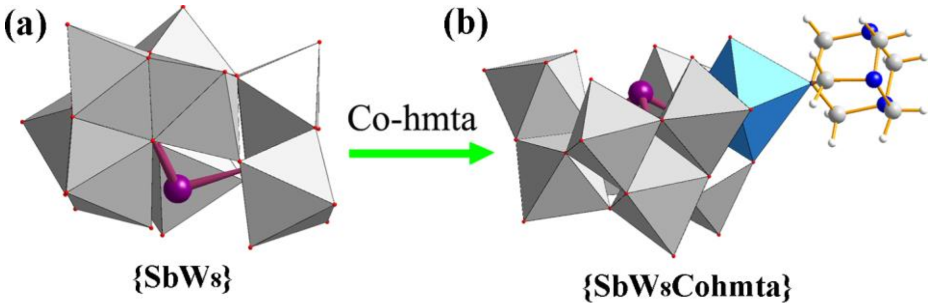

Fig. S4 (a) The tetravalent tungstoantimonate {SbW_8O_{31}} unit and (b) the Co-hmta-substituted {SbW_8O_{31}} subunit in **1** and **2**.

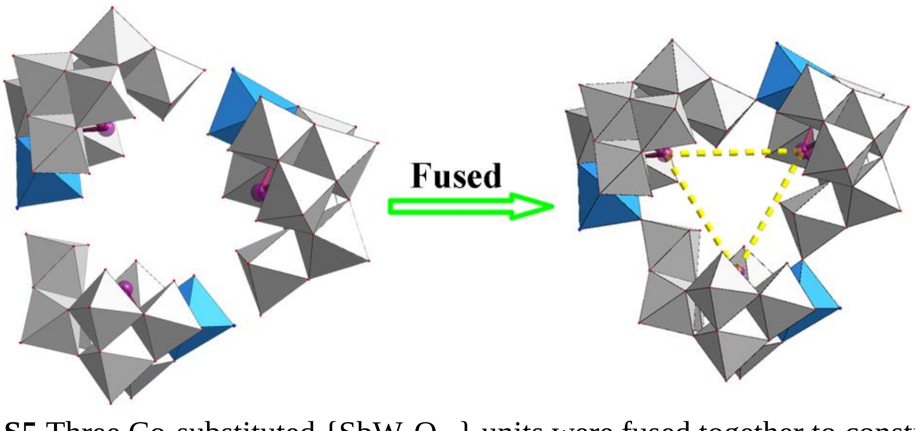

Fig. S5 Three Co-substituted {SbW_8O_{31}} units were fused together to constitute a triangular shell.

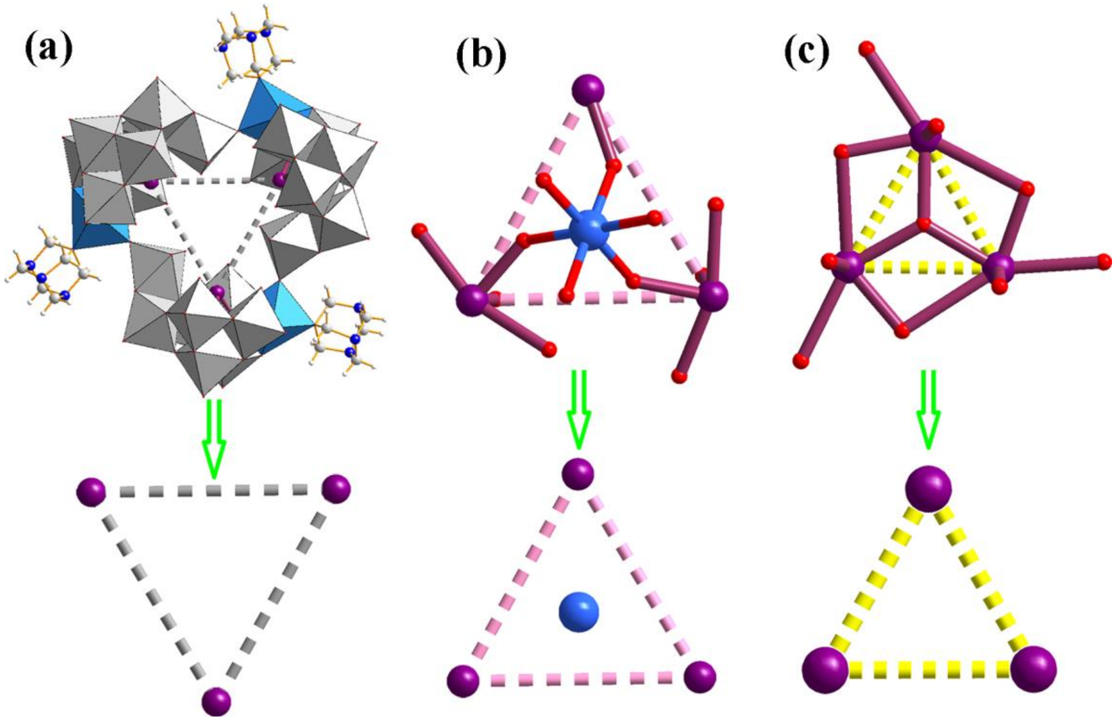

Fig. S6 The triangular groups in the chiral polyoxoanion.

Section 3 Supplementary Physical Characterizations

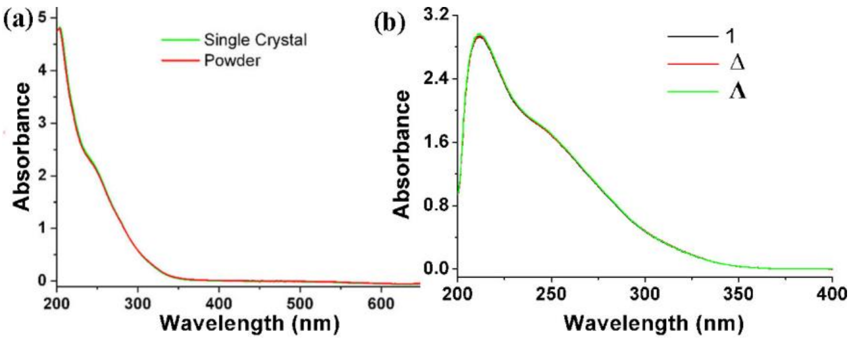

Fig. S7 (a) UV-Vis spectra of **2** for one single-crystal and bulk sample at similar concentrations; (b) UV-Vis spectra of achiral **1**, Δ-**2,** and Λ-**2** in aqueous solution at similar concentrations.

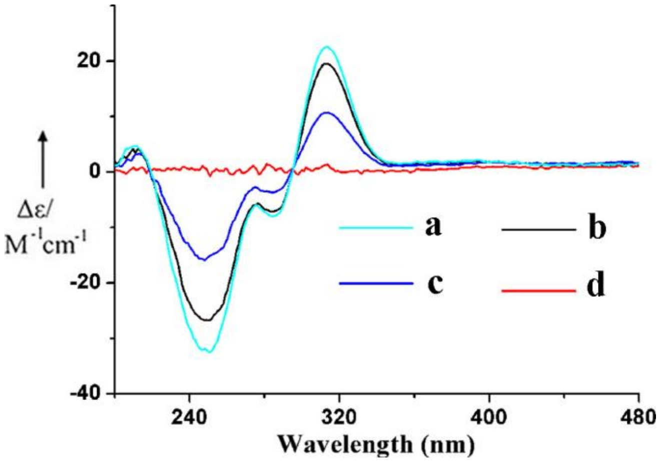

Fig. S8 (a) CD spectra of a chiral single crystal; (b) and (c) powder samples obtained during a fast crystallization process; (d) CD spectrum of the racemic solution after the reaction.

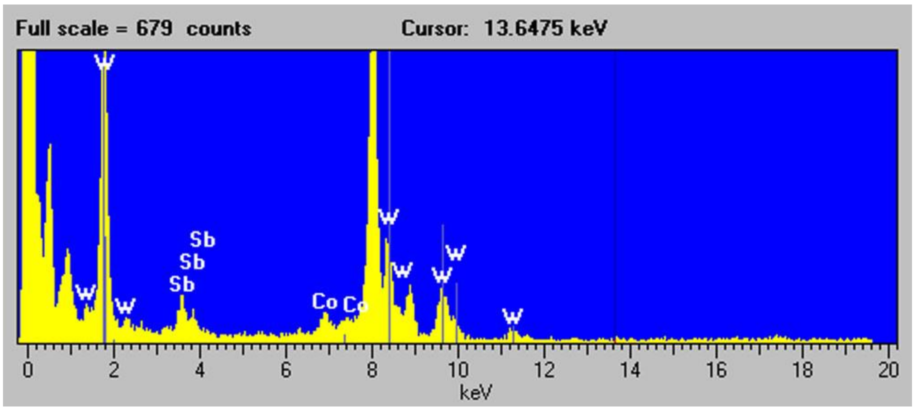

Fig. S9 Energy-dispersive X-ray (EDX) analysis of Δ-**2**.

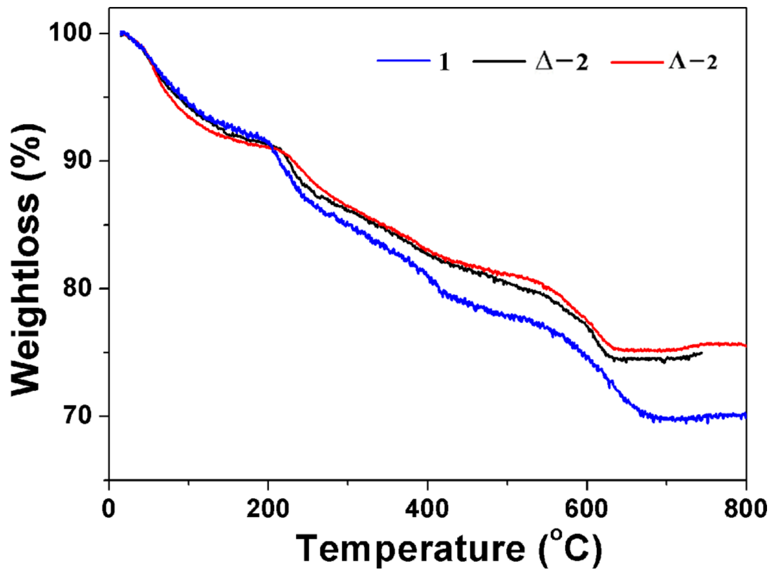

Fig. S10 The TG curve of compounds **1**, Δ-**2**, and Λ-**2**. In these TG curves, there are three continual weight losses in the temperature range of ~25-700 °C. The first weight losses of 8.50% (**1**) and 8.65% (Δ-**2** and Λ-**2)** in the temperature range of ~25-200 °C are attributed to the loss of water molecules counter to the ammonium ions. The weight losses of 14.70% (**1**), 12.05% (Δ-**2**), and 11.20% (Λ-**2)** in the temperature range of ~200-550 °C are attributed to the loss of noncoordinated and coordinated hmta molecules (calcd. 14.78% for **1**, 12.13% for Δ-**2** and Λ-**2**).

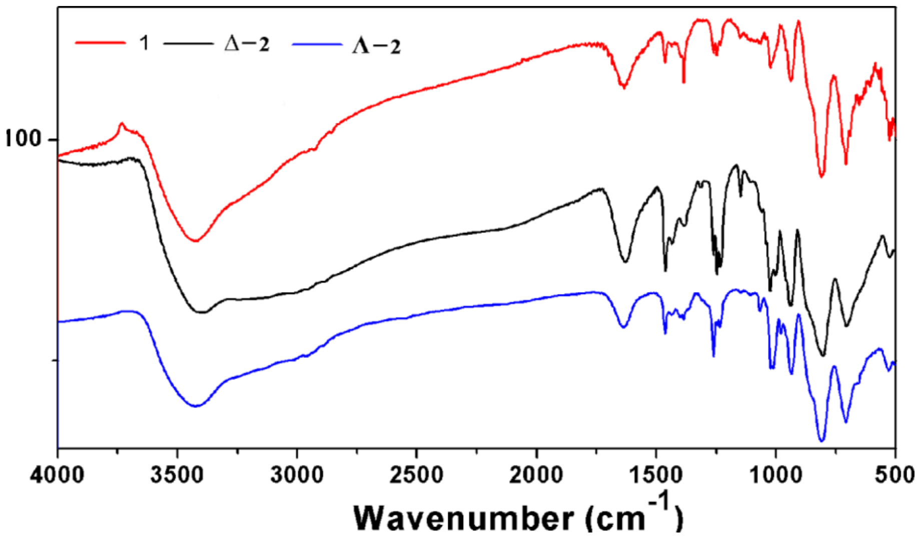

Fig. S11 IR spectra for compounds **1**, Δ-**2**, and Λ-**2**. The IR spectra of Δ-**2** and Λ-**2** are similar to each other, which could help to confirm the structural similarity of these two compounds.

Section 4 Stability Study

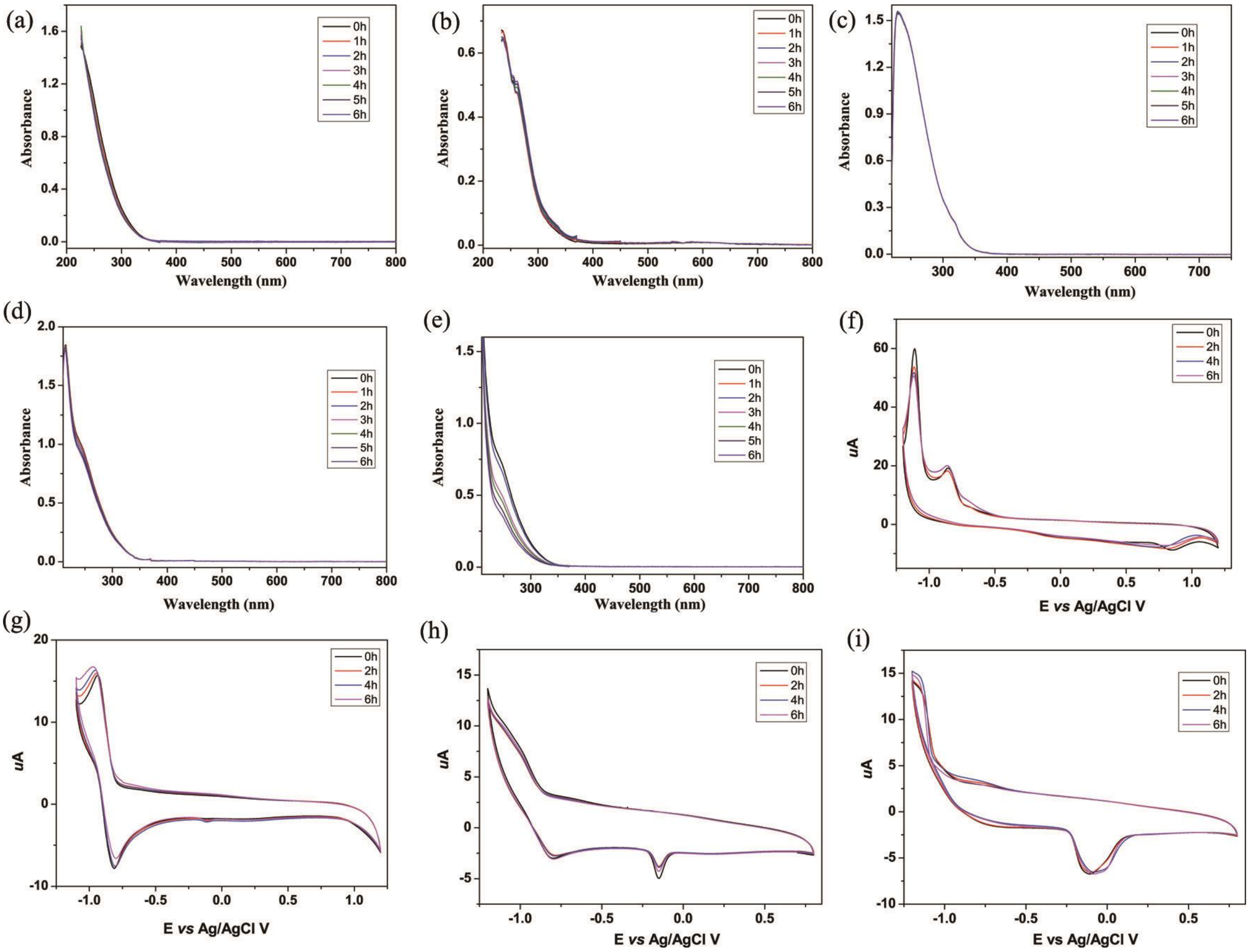

Fig. S12 UV-Vis spectra of **2** in pH 5 (a), pH 6 (b), pH 7 (c), pH 8 (d) and pH 9 (e) buffer solutions. UV-Vis curves were detected every hour for six hours; cyclic voltammograms of **2** (2.5×10^{-4} M) in pH 5 (f), pH 6 (g), pH 7 (h) and pH 8 (i) buffer solutions. The CV curves were detected every two hours for a total of four times in eight hours.

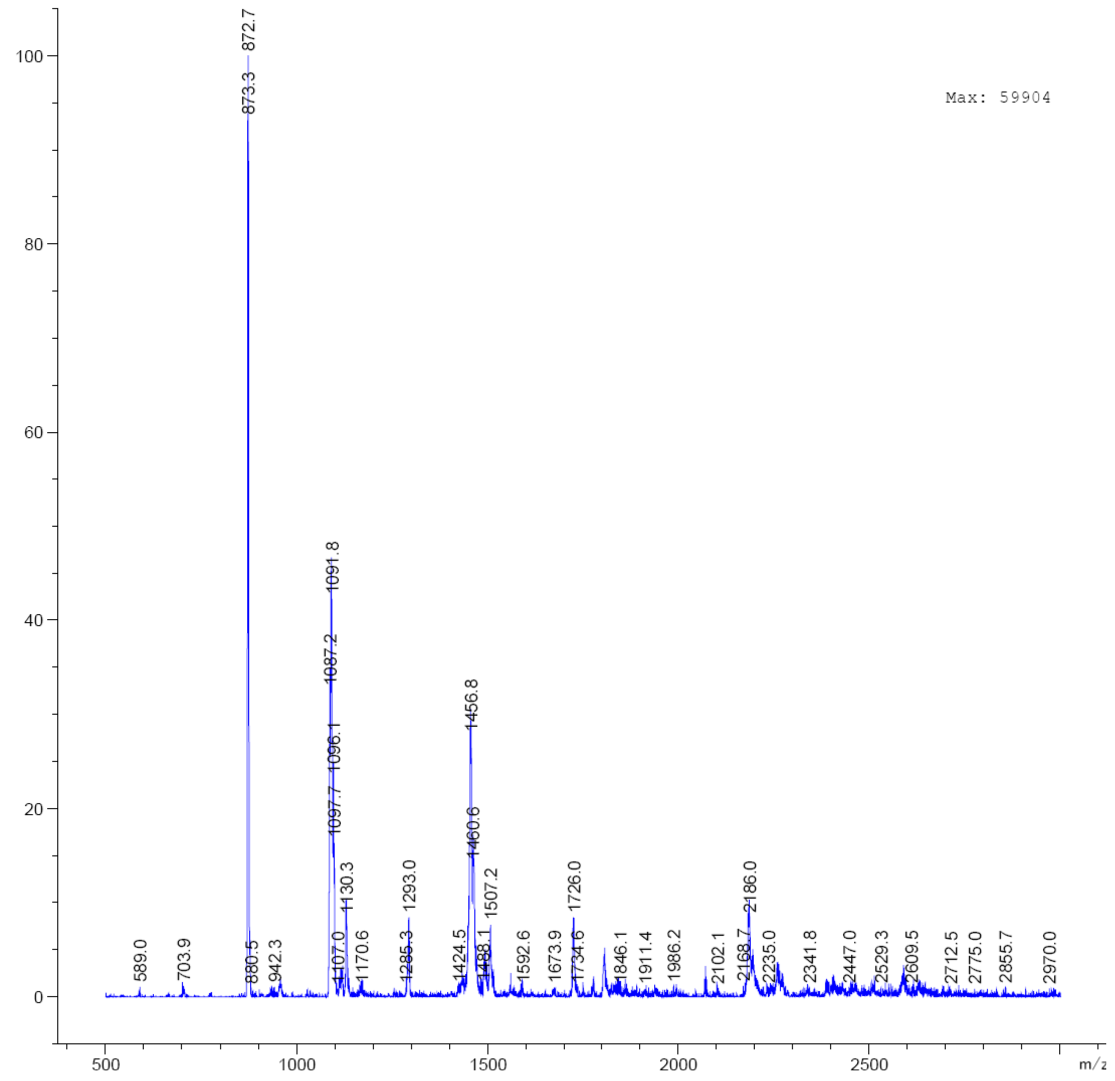

Fig. S13 ESI-MS spectra obtained for **1** dissolved in a pH 7 solution and aged for 24 hours.

Table S2 *M/Z* peak assignments in the ESI-MS spectra of **1**.

Peak assignments	Observed m/z	Calculated m/z
$[Na_3HPOM]^{10-}$	873.3	873.1
$[(Hmta)_6POM]^{8-}(CH_3OH)_3$	1091.8	1091.4
$[K_3(L_2)Co_4P_2W_{18}O_{68}]^{5-}(CH_3OH)_2(DMSO)_2$	1096.7	1096.1
$[H_8POM]^{6-}$-$2H_2O$	1293.0	1293.3
$[(Hmta)_8POM]^{6-}(CH_3CN)_4$	1507.2	1506.9
$[(Hmta)_5Na_4POM]^{5-}(CH_3CN)$	1726.0	1725.5
$[(Hmta)_5NaK_4POM]^{4-}(CH_3CN)CH_3OH$	2186.0	2186.5

Section 5 Anticancer Activity Study

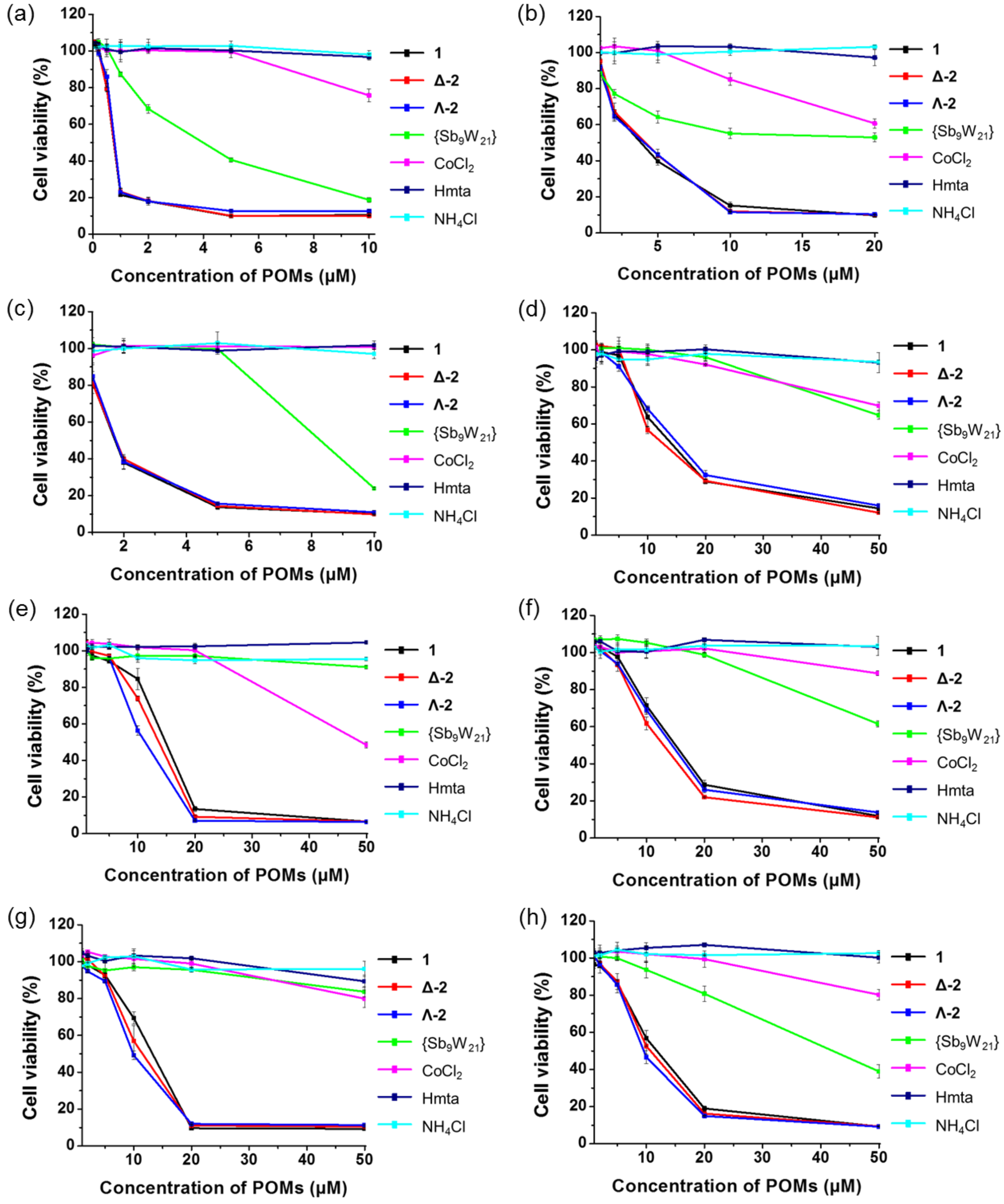

Fig. S14 Cytotoxicity of POMs against (a) A2780, (b) A2780cisR, (c) OVCAR-3, (d) SKOV-3, (e) CT26, (f) HT29, (g) A549, and (i) MCF-7 cells, as determined by the MTS assay after incubation for 72 hours. The concentrations of $CoCl_2$, Hmta, and NH_4Cl were 5-, 10-, and 18-fold greater than that of the POMs, respectively.

Table S3 IC_{50} values (μM) of POMs against SKOV-3, CT26, HT29, A549 and MCF-7 cells after a 72-hour incubation, as determined by MTS assay. Data are expressed as means ± S.D. (n = 3).

	SKOV-3 cells	CT26 cells	HT29 cells	A549 cells	MCF-7 cells
**1**	15.02 ± 0.21	14.72 ± 0.77	15.60 ± 0.78	12.65 ± 0.41	12.24 ± 0.79
**Δ-2**	14.81 ± 0.46	12.98 ± 0.24	13.46 ± 0.41	10.99 ± 1.05	11.14 ± 0.90
**Λ-2**	15.71 ± 0.40	10.65 ± 0.32	14.87 ± 0.21	10.27 ± 0.36	10.01 ± 0.55
$\{Sb_9W_{21}\}$	> 50	> 50	> 50	> 50	43.72 ± 2.44

Table S4 IC_{50} values (μM) of $CoCl_2$, Hmta, and NH_4Cl against A2780, A2780cisR, OVCAR-3, SKOV-3, CT26, HT29, A549 and MCF-7 cells after a 72-hour incubation, as determined by MTS assay. Data are expressed as means ± S.D. (n = 3).

	A2780 cells	A2780cisR cells	OVCAR-3 cells	SKOV-3 cells	CT26 cells	HT29 cells	A549 cells	MCF-7 cells
$CoCl_2$	122.7 ± 3.21	114.6 ± 9.75	> 250	> 250	~250	> 250	> 250	> 250
Hmta	> 500	> 500	> 500	> 500	> 500	> 500	> 500	> 500
NH_4Cl	> 900	> 900	> 900	> 900	> 900	> 900	> 900	> 900

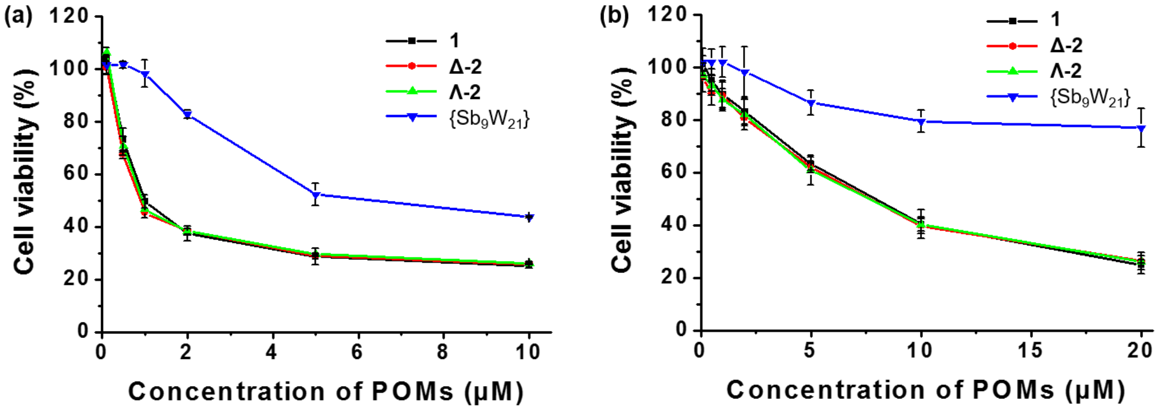

Fig. S15 Cytotoxicity of POMs against (a) A2780 and (b) A2780cisR cells after a 24-hour incubation, as determined by MTS assay.

Table S5 IC_{50} values (μM) of POMs against A2780 and A2780cisR cells after a 24-hour incubation, as determined by MTS assay. Data are expressed as means ± S.D. (n = 3).

	A2780 cells	A2780cisR cells
**1**	1.50 ± 0.10	9.54 ± 1.56
**Δ-2**	1.52 ± 0.10	9.72 ± 1.03
**Λ-2**	1.58 ± 0.20	9.61 ± 0.87
$\{Sb_9W_{21}\}$	8.02 ± 0.20	54.37 ± 4.00

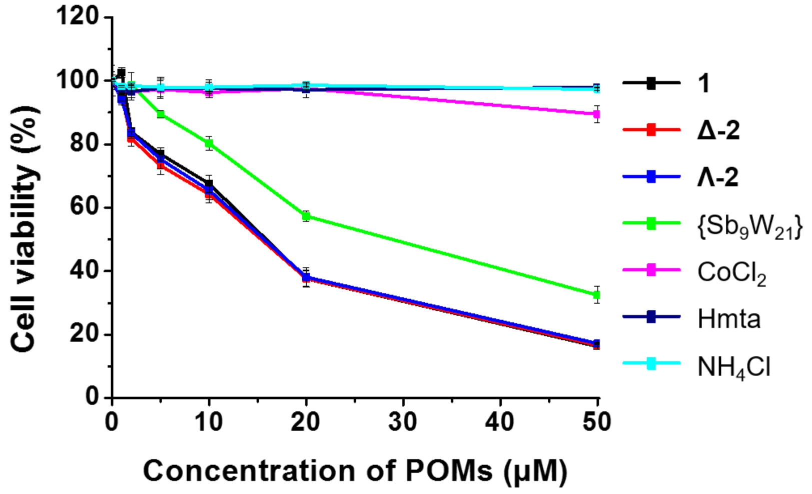

Fig. S16 Cytotoxicity of POMs against HEK-293 cells after a 72-hour incubation, as determined by the MTS assay. The concentrations of $CoCl_2$, Hmta, and NH_4Cl were 5-, 10-, and 18-fold greater than that of the POMs, respectively.

Table S6 IC_{50} values (μM) of POMs, $CoCl_2$, Hmta, and NH_4Cl against HEK-293 cells after a 72-hour incubation, as determined by MTS assay. Data are expressed as means ± S.D. (n = 3).

**1**	**Δ-2**	**Λ-2**	$\{Sb_9W_{21}\}$	$CoCl_2$	Hmta	NH_4Cl
16.17 ± 0.83	16.08 ± 0.50	16.15 ± 1.21	34.47 ± 1.07	>250	> 500	> 900

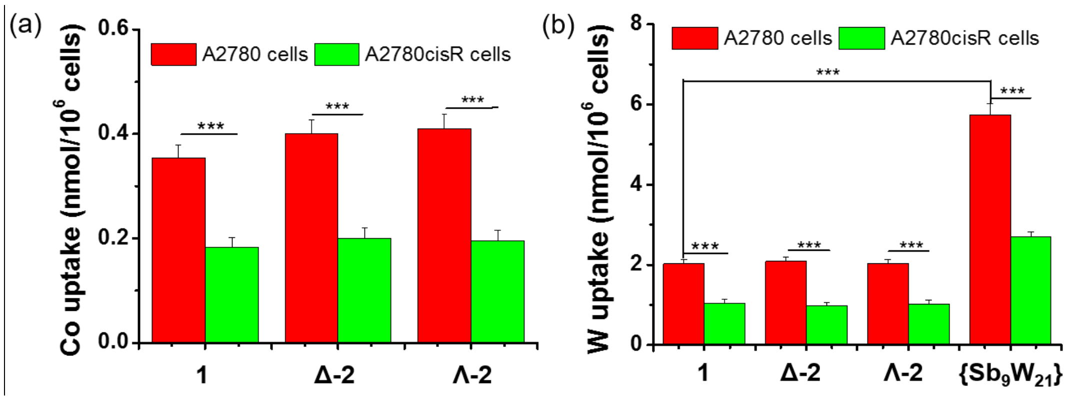

Fig. S17 Uptake of (a) Co and (b) W by A2780 and A2780cisR cells incubated with POMs for 4 hours. Statistical significance: ***$P < 0.001$.

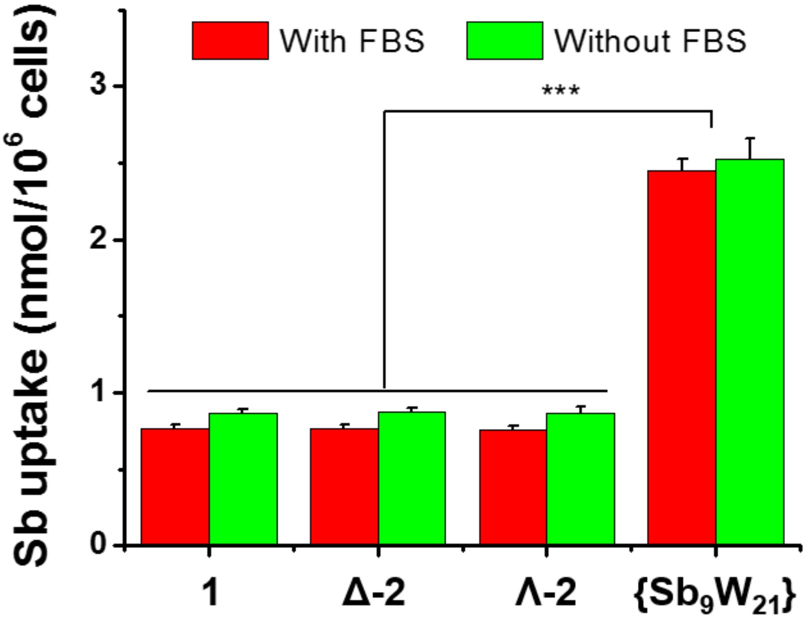

Fig. S18 Uptake of Sb by A2780 cells incubated with POMs for 4 hours in the presence or absence of 10% FBS. Statistical significance: ***$P < 0.001$.

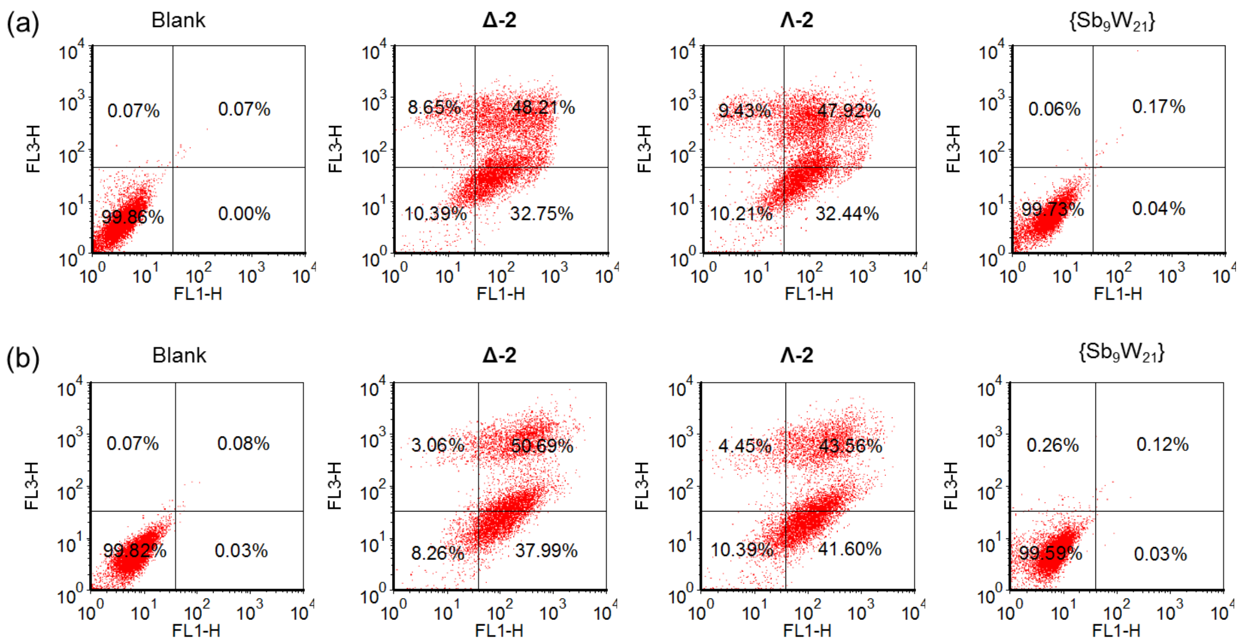

Fig. S19 Annexin V/PI analysis of (a) A2780 and (b) A2780cisR cells after incubation with POMs for 24 hours. The quadrants from lower left to upper left (counter-clockwise) represent healthy, early apoptotic, late apoptotic, and necrotic cells, respectively. The percentage of cells in each quadrant is shown on the graphs.

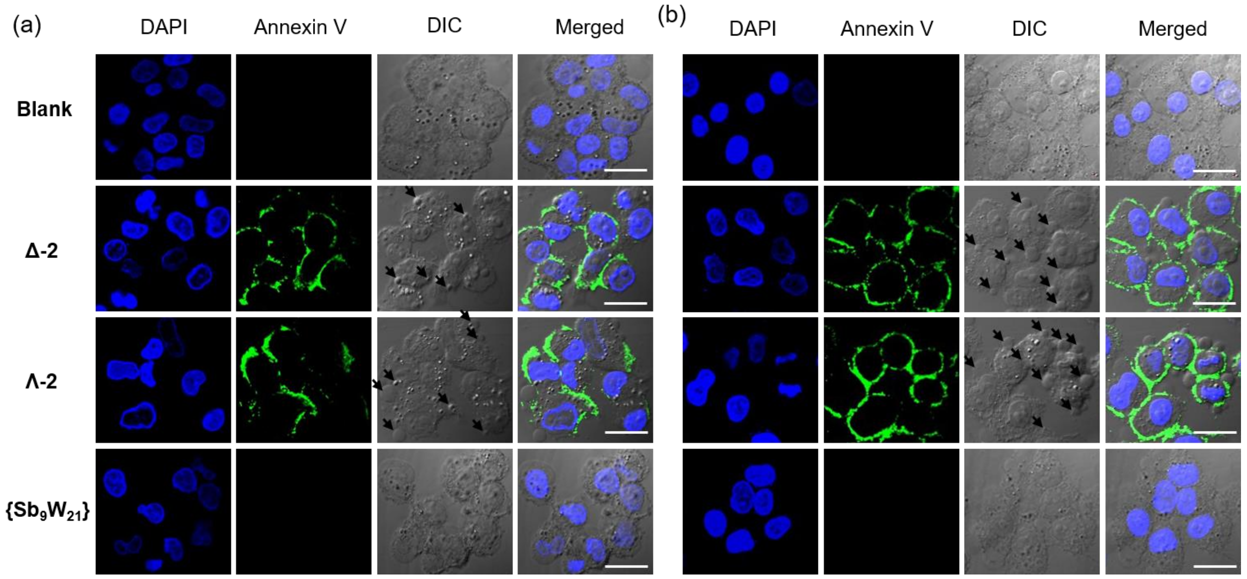

Fig. S20 CLSM images showing cell apoptosis induced by **Δ-2**, **Λ-2**, and $\{Sb_9W_{21}\}$ in (a) A2780 and (b) A2780cisR cells. Scale bars=20 μM.

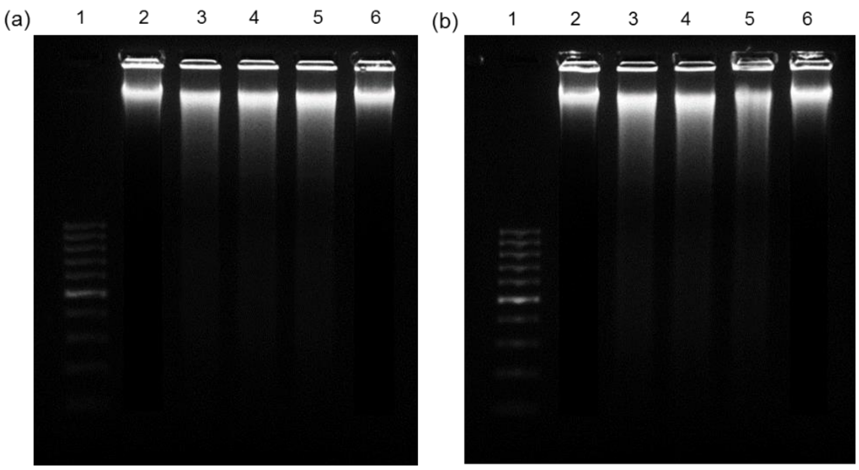

Fig. S21 Analyses of DNA ladders on 2% (w/v) agarose gel at 35 V for 3 hours after DNA extraction from (a) A2780 and (b) A2780cisR cells treated with POMs. Lanes 1-6 show DNA marker, blank, **1**, Δ-**2**, Λ-**2**, and {Sb_9W_{21}}, respectively.

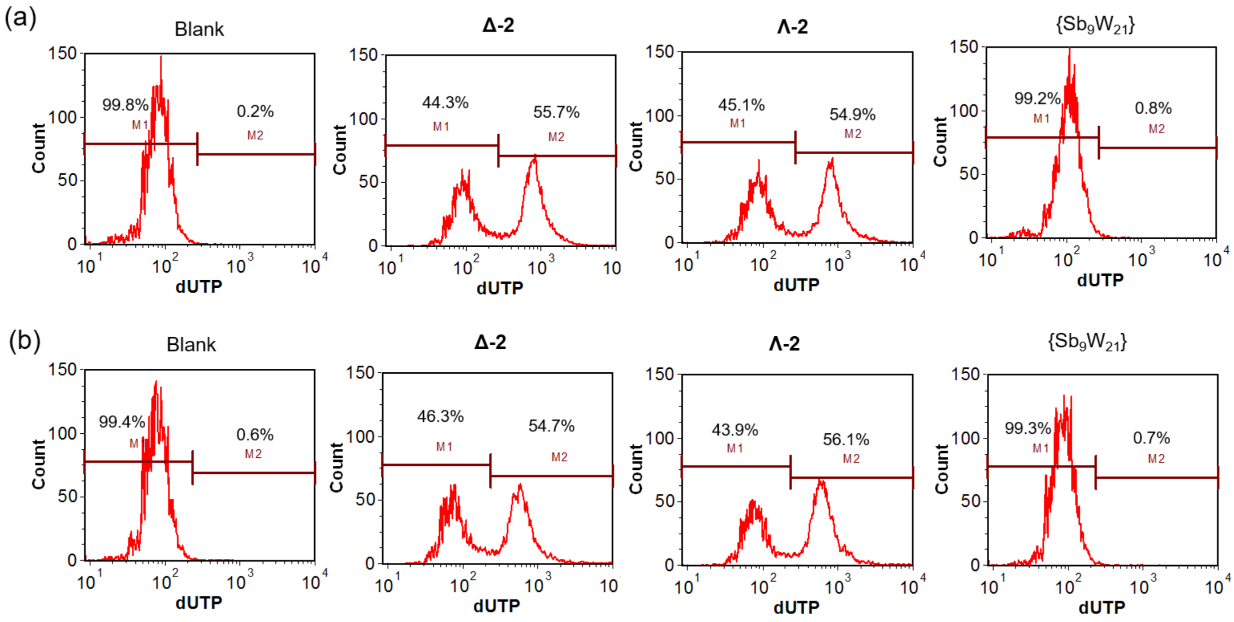

Fig. S22 Flow cytometric analysis of apoptotic and non-apoptotic populations of A2780 and A2780cisR cells treated with POMs by TUNEL assay.

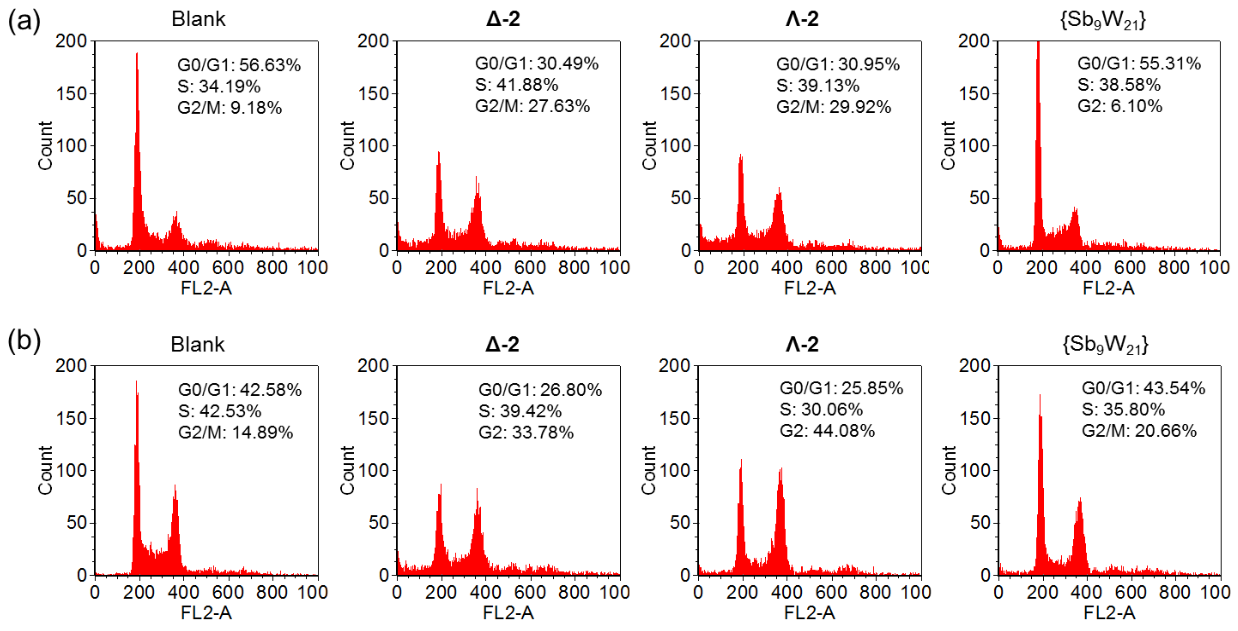

Fig. S23 Cell cycle analysis of (a) A2780 and (b) A2780cisR cells incubated with POMs for 24 hours.

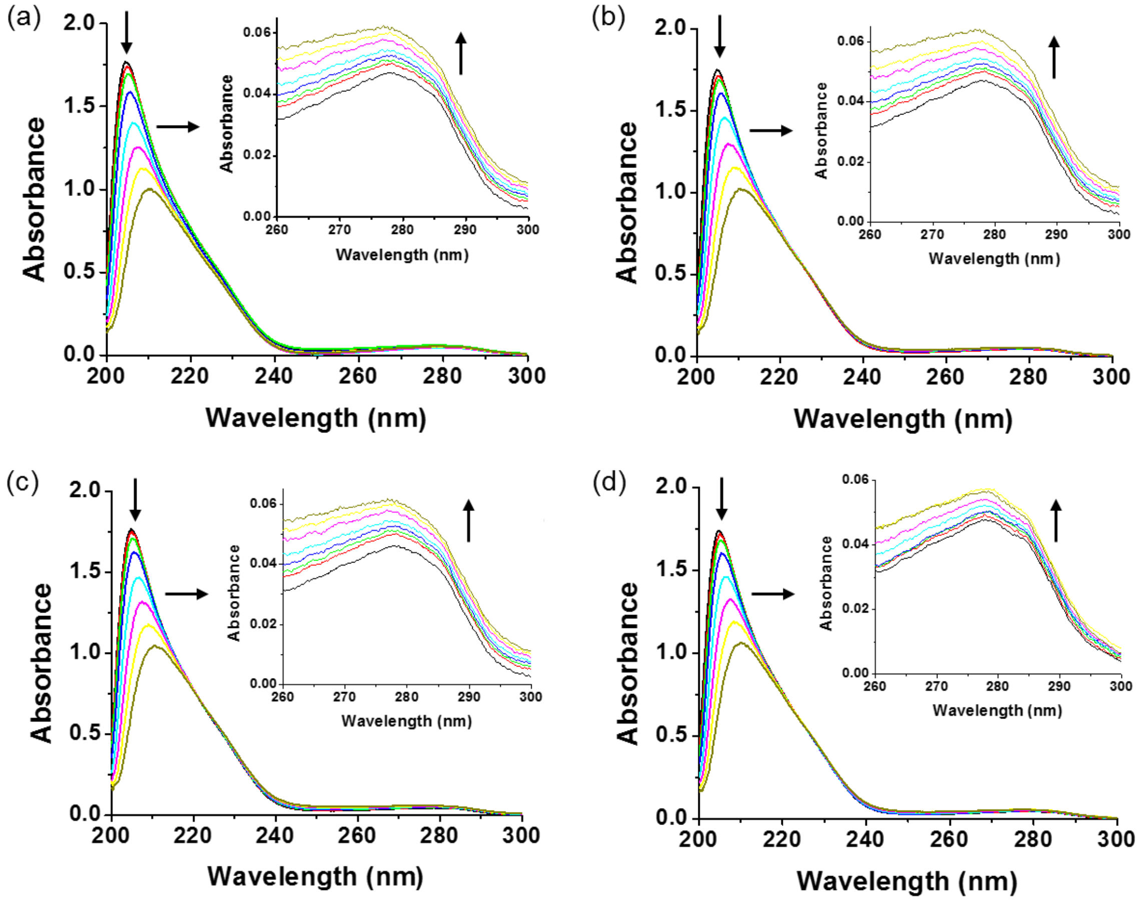

Fig. S24 The UV-vis spectra of BSA after titration with (a) **1**, (b) Δ-**2**, (c) Λ-**2**, and (d) $\{Sb_9W_{21}\}$, respectively.

Table S7 Changes in the UV-vis spectra of BSA after titration with **1**, Δ-**2**, Λ-**2**, and {Sb_9W_{21}}.

	204 nm		280 nm
	ΔA/A	Red-shift	ΔA/A
**1**	-43.3%	5.4	32.8%
Δ-**2**	-41.6%	5.5	35.5%
Λ-**2**	-40.7%	5.9	34.3%
{Sb_9W_{21}}	-38.8%	5.3	18.2%

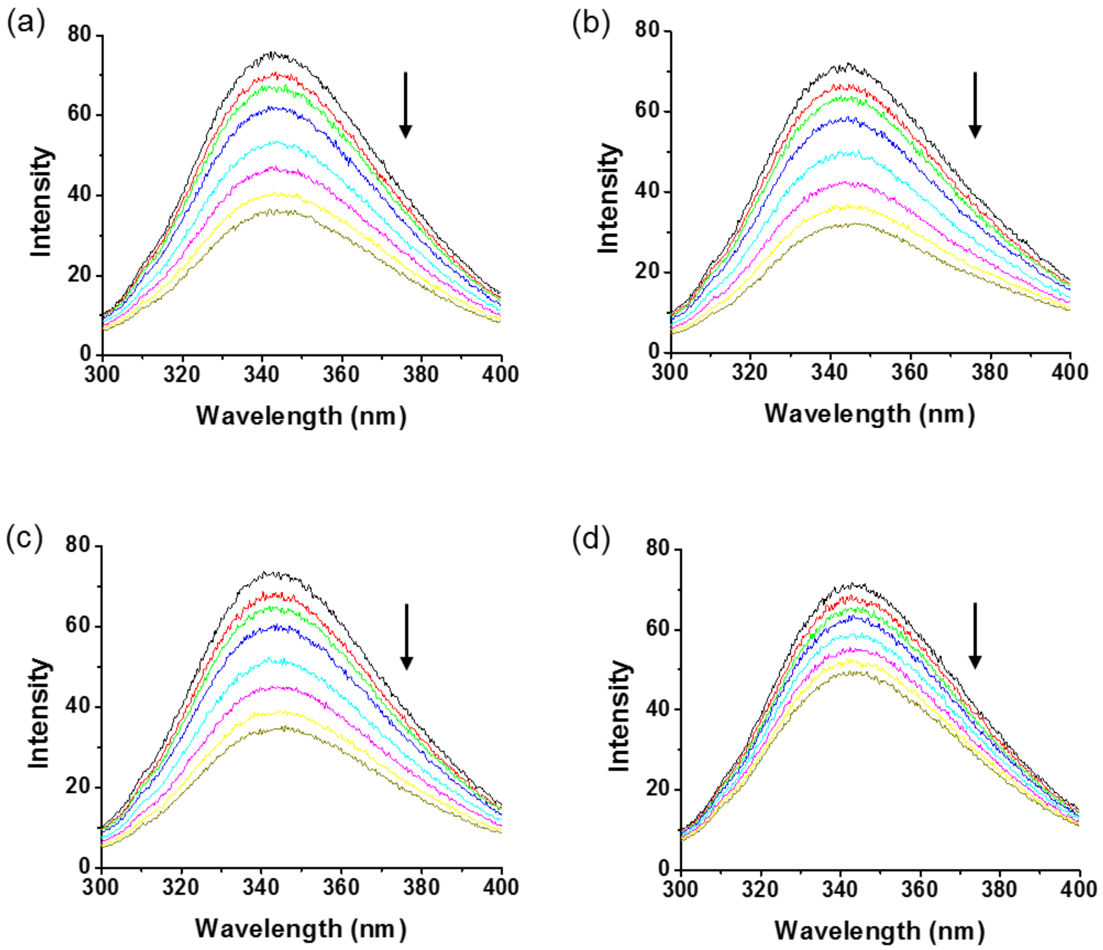

Fig. S25 Fluorescence intensity of BSA after titration with (a) **1**, (b) Δ-**2**, (c) Λ-**2**, and (d) {Sb_9W_{21}}.

Table S8 Stern-Völmer quenching constants of the POM-BSA systems.

	Linear regression equation	R	K_{sv} (L/mol)	K_q [L/(mol s)]
**1**	$F_0/F= 1.01 + 1.02\times10^5[Q]$	0.9991	$(1.02 \pm 0.03)\times10^5$	$(1.02 \pm 0.03)\times10^{13}$
**Δ-2**	$F_0/F= 1.01 + 1.18\times10^5 [Q]$	0.9987	$(1.18 \pm 0.21)\times10^5$	$(1.18 \pm 0.21)\times10^{13}$
**Λ-2**	$F_0/F= 1.01 + 1.11\times10^5 [Q]$	0.9991	$(1.11 \pm 0.89)\times10^5$	$(1.11 \pm 0.89)\times10^{13}$
$\{Sb_9W_{21}\}$	$F_0/F= 1.03 + 0.45\times10^5 [Q]$	0.9986	$(0.45 \pm 0.06)\times10^5$	$(0.45 \pm 0.06)\times10^{13}$

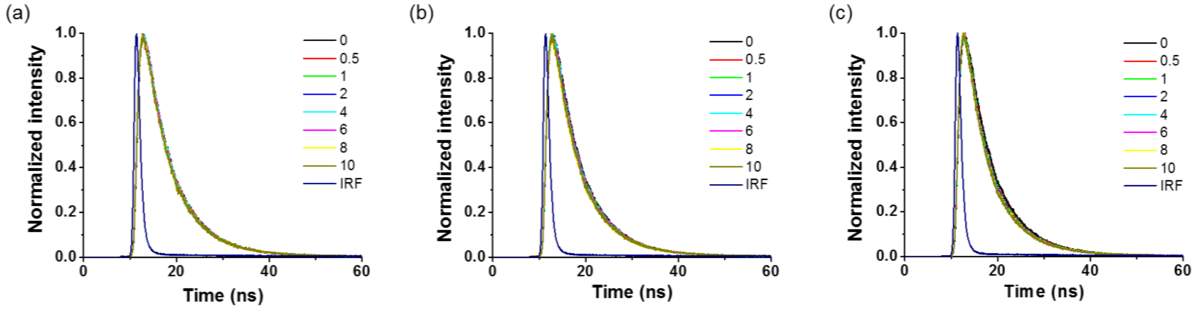

Fig. S26 Time-resolved fluorescence decay traces of BSA titrated with (a) Δ-**2**, (b) Λ-**2**, and (c) $\{Sb_9W_{21}\}$. The molar ratio of POMs to BSA was 0-10.

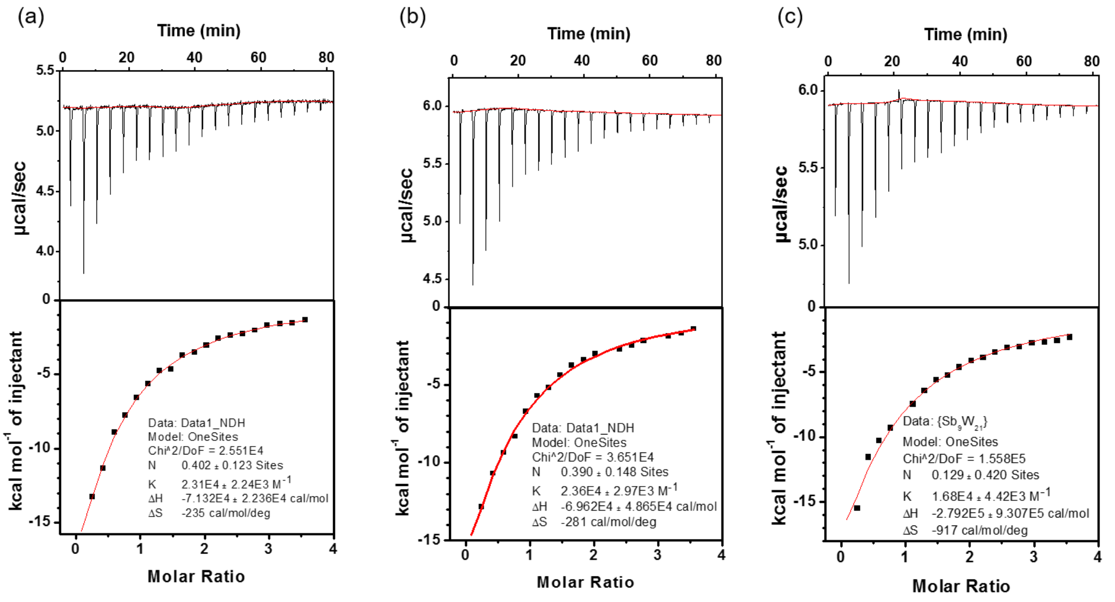

Fig. S27 Calorimetric data for the titration of BSA with (a) Δ-**2**, (b) Λ-**2**, and (c) $\{Sb_9W_{21}\}$. The binding isotherm (heat change vs POM/BSA molar ratio) was obtained from the integration of raw data and fitted to a "one-site" model.
